# Modeling Xanthophyll Excited States via Cost-Effective Quantum Chemistry methods and Property-Based Diabatization

**DOI:** 10.1021/acs.jctc.6c00637

**Published:** 2026-06-20

**Authors:** Amanda Arcidiacono, Valentino Martini, Lorenzo Cupellini, Laura Pedraza-González

**Affiliations:** Dipartimento di Chimica e Chimica Industriale, 9310 Università di Pisa, via G. Moruzzi 13, Pisa 56124, Italy

## Abstract

Xanthophyll carotenoids play essential roles in photosynthetic light harvesting and photoprotection in biological systems, yet the accurate description of their excited states at a feasible computational cost remains challenging due to their extended π-conjugation and multireference character. Here, we compare the DFT/MRCI, FOMO–CI, and mixed-reference spin-flip TDDFT (MRSF-TDDFT) descriptions of the excited states for a representative set of xanthophylls. We analyze vertical excitation energies and potential energy profiles along the bond-length alternation coordinate, adopting a multiple-property-based diabatization (MPD) scheme to consistently characterize electronic states across methods. We find a remarkably consistent description of the low-lying excited states and their potential energy curves across the methods. DFT/MRCI and the semiempirical FOMO–CI exhibit closely aligned behavior across xanthophylls, while the performance of MRSF-TDDFT depends on the choice of exchange-correlation functional. Overall, our calculations suggest that the covalent 2A_*g*_^–^ state lies below the ionic 1B_*u*_^+^ at the Franck–Condon point. Our analysis provides general insight into the electronic structure of xanthophylls, including the interplay between covalent and ionic configurations induced by structural distortions and environmental effects. The MPD framework enables a direct comparison of state character, highlighting the similarities between methods and clarifying the origin of remaining differences. This work establishes a consistent framework for understanding xanthophyll photophysics and offers practical guidance for modeling their excited states with cost-effective methods.

## Graphical abstract

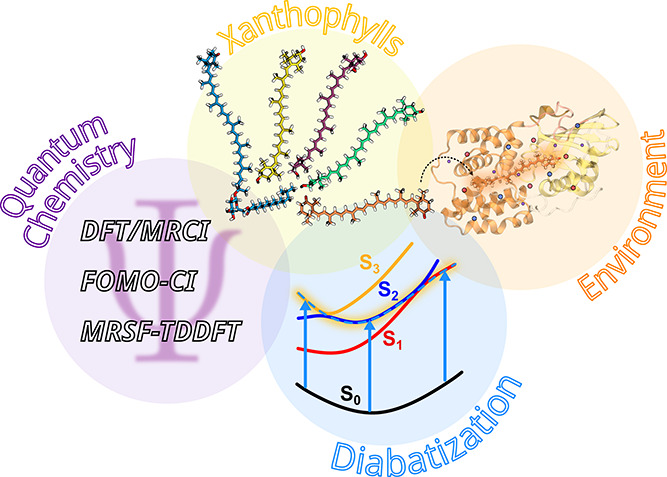

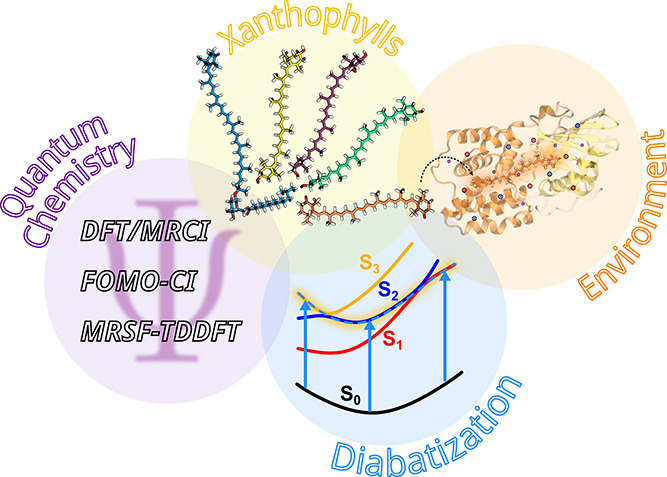

## 1 Introduction

Xanthophylls are a major subclass of carotenoids, characterized by oxygenated functional groups such as hydroxyl, keto, or epoxide moieties (Figure 1a). They are ubiquitous in nature and embedded in a wide range of biological assemblies. In photosynthesis, they fulfill multiple functions, such as light harvesting,^1–4^ excitation-energy transfer, and especially photoprotection, including nonphotochemical quenching.^5–11^ The versatility of xanthophyll function is directly linked to the properties of their low-lying excited states, including their energetic ordering, coupling, and relaxation pathways, which are highly sensitive to molecular structure and environment.^12–15^

**Figure 1:**
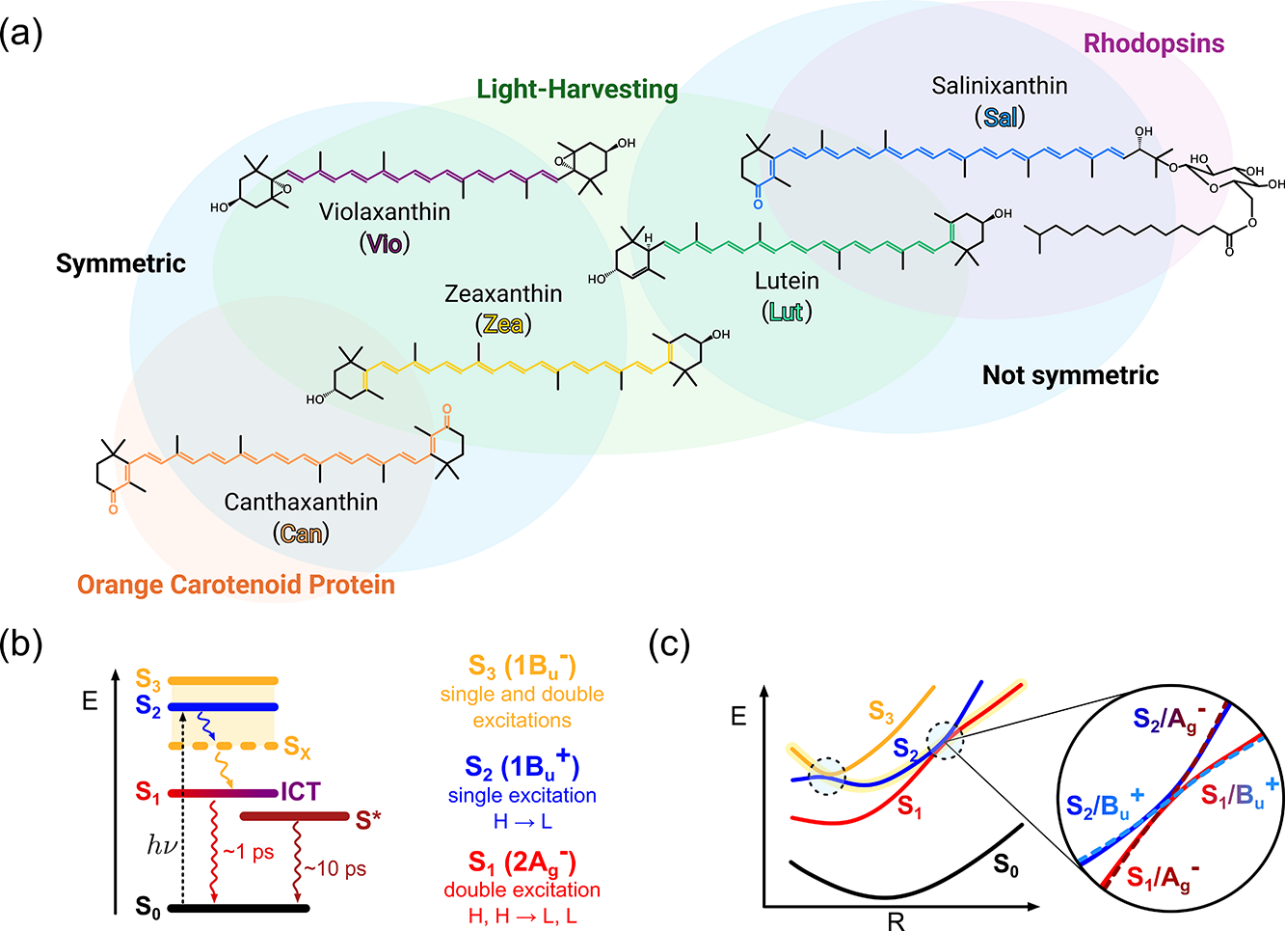
Structural diversity and excited-state landscape of xanthophyll carotenoids. (a) Chemical structures of representative xanthophylls investigated in this work, spanning diverse chemical functionalities (hydroxyl, keto, and epoxide substituents). The conjugated polyene backbone is highlighted. Pigments are classified according to molecular symmetry (symmetric vs asymmetric) and their predominant protein environments. (b,c) Schematic representation of the electronic structure of xanthophylls.^16,17^ (b) Excited-state dynamics within the low-lying manifold, including a simplified characterization of state character.^18–22^ (c) Conical intersections between the low-lying excited states (S_1_/S_2_ and S_2_/S_3_),^16,23^ highlighting the exchange of electronic character at the S_1_/S_2_ intersection. H: HOMO, L: LUMO.

The extended conjugated polyene backbone of carotenoids gives rise to a characteristic manifold of low-lying excited states.^13,19,24^ This manifold comprises a dark 2A_*g*_^–^ state of pronounced double excitation character lying below the optically bright 1B_*u*_^+^ state,^13,14,25–30^ but a number of additional and controversial dark states have been proposed^19^ (Figure 1b). Crossings between these states, as well as with higher-lying singlets, govern ultrafast internal conversion and mediate the exchange of electronic character (Figure 1c). Functional-group substitution and torsional flexibility further modulate the electronic landscape. Excited states of carotenoids are also especially sensitive to collective single- and double-bond stretching modes of the conjugated backbone.^24^ In polar solvents and protein environments, additional electrostatic and steric effects may stabilize intramolecular charge-transfer (ICT) states, further reshaping the excited-state landscape.^19,22^ A reliable theoretical description must therefore capture states of fundamentally different character, their geometry-dependent state mixing, and environment-induced polarization effects within systems of biologically relevant size.

Despite substantial methodological progress, achieving accurate excited-state predictions at a feasible computational cost remains challenging for extended conjugated systems such as carotenoids. Multireference approaches based on complete active space self-consistent field (CASSCF) theory provide a rigorous framework to describe the interplay between covalent and ionic configurations underlying the 2A_*g*_^–^ and 1B_*u*_^+^ states, as long as dynamic correlation is incorporated.^14,15,30–35^ Second-order multireference perturbation theories, such as CASPT2 ^36,37^ and NEVPT2 ^38–41^ provide a way to include dynamic correlation. However, a balanced and converged treatment requires very large active spaces,^42,43^ rendering such calculations rapidly computationally prohibitive. Although approximate solvers for the CASSCF wave function, such as the density matrix renormalization group (DMRG), have significantly extended the accessible active-space size,^14,15,32,34^ fully ab initio descriptions of carotenoids embedded in protein environments are still not routinely feasible.

Density functional theory (DFT) methods offer more affordable alternatives. However, linear-response time-dependent DFT (TDDFT)^44^ fails to describe the 2A_*g*_^–^ state due to its pronounced double-excitation character.^45,46^ Spin-flip TDDFT^47^ partially solves this deficiency by accessing an alternative excitation manifold, and has been shown to provide reasonable geometries for the 2A_*g*_^–^ state.^15,48^ However, its accuracy for excitation energies remains limited, possibly due to spin contamination as well as to the choice of exchange-correlation functional.^15,48^ The mixed-reference spin-flip formulation (MRSF-TDDFT) alleviates the spin contamination problem and augments the effective excitation space, which enables a more balanced treatment of strongly correlated excited states.^49–52^ Nevertheless, the dependence on the exchange-correlation functional remains. While this method has been used for polyenes, systematic benchmarks for carotenoids are missing.

DFT/MRCI has emerged as a practical compromise between accuracy and computational efficiency for multireference systems.^53,54^ DFT/MRCI combines Kohn–Sham orbitals with a multireference configuration-interaction treatment. By introducing configuration interaction on top of a DFT reference, it grants access to excitation manifolds beyond the linear-response regime at substantially lower cost than fully ab initio multireference approaches. However, DFT/MRCI introduces parameters to avoid double counting of dynamic correlation. Its accuracy is thus tied to the chosen parametrization, namely the DFT/MRCI Hamiltonian. Earlier parameter sets were successfully applied to carotenoids and enabled the description of both dark and bright states, but displayed systematic biases in their relative energetic ordering and state character.^45,46,55–57^ The recent R2022 Hamiltonian^58^ was developed to improve robustness and transferability across chemical space. Whether this improvement extends to xanthophylls remains to be established.

In parallel, multireference semiempirical methods have been widely applied to carotenoids owing to their favorable computational scaling.^6,7,31,59–63^ This strategy combines semiempirical Hamiltonians with a configuration-interaction (CI) treatment of the excited states, thereby retaining a multiconfigurational description at substantially reduced cost. Full CI calculations within active spaces constructed from MNDO molecular orbitals (MNDO-CAS-CI) have been employed to model carotenoid excited states,^7,61^ and related MNDO- and INDO/S-based CI frameworks have been used to investigate excited-state dynamics in extended conjugated systems.^17,64^ More recently, a floating occupation molecular orbital CI (FOMO–CI) strategy^65–67^ has enabled excited-state calculations of carotenoids in various environments,^16,21,23,68–71^ including excited-state quantum mechanics/molecular mechanics (QM/MM) optimizations. The FOMO–CI strategy allows achieving a more balanced description of excited states, and the specific reparametrization for Lutein^16^ of the underlying AM1 Hamiltonian achieved an improved agreement with experiments. Nonetheless, the transferability of this method across xanthophylls has not been systematically assessed.

Extended xanthophylls provide a demanding test system for excited-state methodologies, as they combine pronounced multireference character, geometry-driven state reordering, and sensitivity to the environment within systems of considerable size. In particular, (avoided) crossings along the bond length alternation (BLA) coordinate (Figure 1c) render adiabatic state identities method-dependent, complicating direct comparison of excited-state characters across electronic structure approaches. A consistent, property-based framework for tracking state character is thus required to understand the electronic structure description.

To address this challenge, we introduce a diabatization scheme based on multiple properties^72^ that constructs smoothly varying diabatic states along the BLA pathway. By enforcing the continuity of physically meaningful observables, this framework enables consistent tracking of bright and dark states across geometries and methodologies, providing a unified basis for comparing energetic ordering, transition dipole moments, and mixing patterns.

Within this framework, we compare three complementary approaches for modeling xanthophyll photophysics: DFT/MRCI with the R2022 parametrization,^54,58^ the semiempirical FOMO–CI method,^65–67^ and MRSF-TDDFT^49^ employing a representative set of recently developed exchange-correlation functionals. We investigate five structurally diverse xanthophylls, spanning symmetric and asymmetric substitution patterns (Figures 1a and S1). Beyond vertical excitation energies at the Franck–Condon geometry, we analyze the evolution of the three lowest excited states along the BLA coordinate as a probe of multireference character and geometry-driven mixing. Furthermore, we examine intramolecular charge-transfer signatures in asymmetric xanthophylls in vacuum and assess the emergence of ICT states in the symmetric canthaxanthin within the environment of the Orange Carotenoid Protein.^73,74^

We find that the cost-effective methods investigated in this work provide a consistent and accurate description of the electronic state manifold of xantophylls, although MRSF-TDDFT results strongly depend on the choice of the DFT functional. Furthermore, our results support that the dark 2A_*g*_^–^ state lies below 1B_*u*_^+^ also at the Franck–Condon geometry. Overall, our work identifies computationally feasible and physically reliable strategies for modeling xanthophyll excited states in complex biological environments.

## 2 Methods

### 2.1 Multiple Property-Based Diabatization

In order to meaningfully analyze the electronic states of xanthophylls, it is advantageous to adopt a diabatic representation, in which the electronic character evolves smoothly with molecular geometry. The diabatic representation can be defined as follows (eq 1):

$$\left|\phi_{k}\rangle =\sum_{i=1}^{n_{s}}U_{ki}|\psi_{i}\right\rangle$$ 1

where {ψ_*i*_}_*i* = 1_^*n*_s_^ are the adiabatic states and {ϕ_*k*_}_*k* = 1_^*n*_s_^ are the (quasi)-diabatic states.

There exist many approaches for obtaining diabatic states.^75^ Among them, property-based diabatization strategies have the advantage of not being dependent on the electronic structure method. However, diabatization approaches based on a single property face degeneracy issues, especially when multiple states are considered.^76^ Here, we follow the strategy outlined in ref 72, where multiple properties are combined in the construction of the adiabatic-to-diabatic (ATD) transformation. This approach, termed multiple-property diabatization (MPD), is outlined as follows.

Let us consider *n*_p_ properties (i.e., observables) for *n*_s_ states. For each property, the corresponding operator can be written in the adiabatic basis as (eq 2):

$$A_{p}^{ij}=\langle \psi_{i}|O_{p}|\psi_{j}\rangle$$ 2

The set of matrices $\{A_{p}\in R^{n_{s}\times n_{s}}{\}}_{p=1}^{n_{p}}$ thus represents the properties in the *n*_*s*_-dimensional adiabatic basis. Let us now express the same operators in a *n*_s_-dimensional basis that we can consider diabatic, $\{D_{p}\in R^{n_{s}\times n_{s}}{\}}_{p=1}^{n_{p}}$ . The unitary ATD transformation matrix **U** then fulfills the following relations (eq 3):

$$\begin{array}{ll} D_{p}=U^{†}A_{p}U, & \forall p \end{array}$$ 3

The best approximation to the matrix **U** can be obtained by minimizing the Frobenius norm of the following residual (eq 4):

$$E(U{)}^{2}=\sum_{p}\omega_{p}||A_{p}U-{UD}_{p}|{|}^{2}$$ 4

where the sum *p* runs on the different properties, and ω_p_ are weights that can be applied to the different properties. The residual can be written as (eq 5):

$$\begin{array}{rl} E(U{)}^{2} & =\underset{p}{\sum }\omega_{p}\mathrm{Tr}[(A_{p}U-{UD}_{p}{)}^{†}(A_{p}U-{UD}_{p})]\\ & =\underset{p}{\sum }\omega_{p}\mathrm{vec}(U{)}^{†}K_{p}^{†}K_{p}\mathrm{vec}(U)\\ & =\mathrm{vec}(U{)}^{†}M\mathrm{vec}\left(U\right) \end{array}$$ 5

where $M=\sum_{p}\omega_{p}K_{p}^{†}K_{p}$ . Here, $\mathrm{vec}(U)\in R^{n_{s}^{2}}$ is the vectorization of **U**, and **K**_p_ is a *n*_s_^2^ × *n*_s_^2^ matrix given by (eq 6):

$$K_{p}=A_{p}⊗1-1⊗D_{p}$$ 6

where “⊗” denotes the Kronecker product and **1** is the *n* × *n* identity matrix. The minimization has to be carried out with the constraint vec­(**U**)^†^vec­(**U**) = **1**, as **U** is required to be unitary. This leads to determining vec­(**U**) by solving the following eigenvalue problem (eq 7):

Mvec(U)=λvec(U) 7

The transformation matrix U* is then determined by reshaping the eigenvector vec­(U*) associated with the lowest eigenvalue. The eigenvalue problem described above does not ensure that U* is a unitary matrix. However, a matrix minimizing the residuals in eq 4 should be close to unitary. The closest unitary matrix can be determined by means of a singular value decomposition (eq 8):

$$U^{*}=L\Sigma R^{†}$$ 8

and then calculating **U** = **L** **R** ^†^, which is guaranteed to be unitary.

This multiproperty diabatization offers several advantages. First, using multiple properties to characterize the states increases the likelihood of distinguishing multiple electronic states and lifting degeneracies, solving the problematic cases that occur with other property-based strategies.^76^ In practice, if enough properties are considered, there is a unique matrix **U** minimizing the residual eq 4. This can be verified by inspecting the gap between the first and second eigenvalues of **M**.

Importantly, if a unique unitary matrix **U** is found, the solution also ensures that the rows of the transformation matrix have a consistent sign. In fact, if a single reference diabatic state changes sign, then the corresponding row and column in **D**_p_ would change as well, determining a new solution to the eigenvalue problem. Analogously, if any adiabatic state changes sign, a new solution will be obtained. Hence, consistent signs are obtained when the transformation is used to obtain the diabatic Hamiltonian as (eq 9):

$$H^{d}=U^{†}H^{a}U$$ 9

Here, **H** ^*a*^ is a diagonal matrix of elements $H_{i}^{a}=\left\langle \psi_{i}\right|Ĥ\left|\psi_{i}\right\rangle$ , and **H** ^*d*^ is a nondiagonal matrix of elements $H_{kl}^{d}=\left\langle \phi_{k}\right|Ĥ\left|\phi_{l}\right\rangle$ . The off-diagonal elements of **H** ^*d*^, the diabatic couplings, have a consistent sign if **U** is unique.

To ensure that enough properties are taken into account in the diabatization, we propose the Mulliken atomic populations of each atom *a* as observables. The matrix elements of these operators are the Mulliken state or transition populations (eq 10):

$$q_{ij}^{a}=\left\langle \psi_{i}\right|{\mathrm{q̂}}_{a}\left|\psi_{j}\right\rangle =\underset{\mu \in a}{\sum }\underset{\nu }{\sum }P_{\mu \nu }^{ij}S_{\nu \mu }$$ 10

where **P** ^*ij*^ is the transition density matrix between two states *i* and *j* when *i*≠*j*, and **P** ^*ij*^ = **P** ^*i*^ is the density matrix of a state *i* when *i* = *j*. μ and ν are indices running over atomic orbitals localized on atom a. Mulliken populations are a convenient choice because they directly reflect the density matrix while remaining rotationally invariant. The density matrix itself does not share these invariance properties, which prevents its direct use in these transformations.

The diabatic properties, **D**_*p*_, are determined by choosing an appropriate reference geometry where the adiabatic and diabatic states coincide. In ref 72, the reference properties were constructed from isolated monomers of a dimer, a strategy that cannot be applied here. Here, we chose as a reference the adiabatic properties at the ground-state equilibrium geometry, where we assume that adiabatic and diabatic states coincide. In Section S10 of the Supporting Information, we assess the effect of choosing different geometries as references for the diabatization.

For any geometry of a given xanthophyll in a vacuum, we follow the strategy illustrated in Figure 2a. For the gas-phase diabatization, we found that restricting the procedure to the first four electronic states provides the most reliable results, even though a more rigorous treatment would, in principle, require the inclusion of additional states. The higher-lying states are in fact very close in energy, similar in character, and already significantly mixed at the reference geometry, which limits their usefulness in the diabatization, as shown in the analysis in Section S10 of the Supporting Information. However, at least in the gas phase, the mixing between the S_0_-S_3_ states and higher energy states (which are also well separated in energy) is negligible in most of the cases.

**Figure 2:**
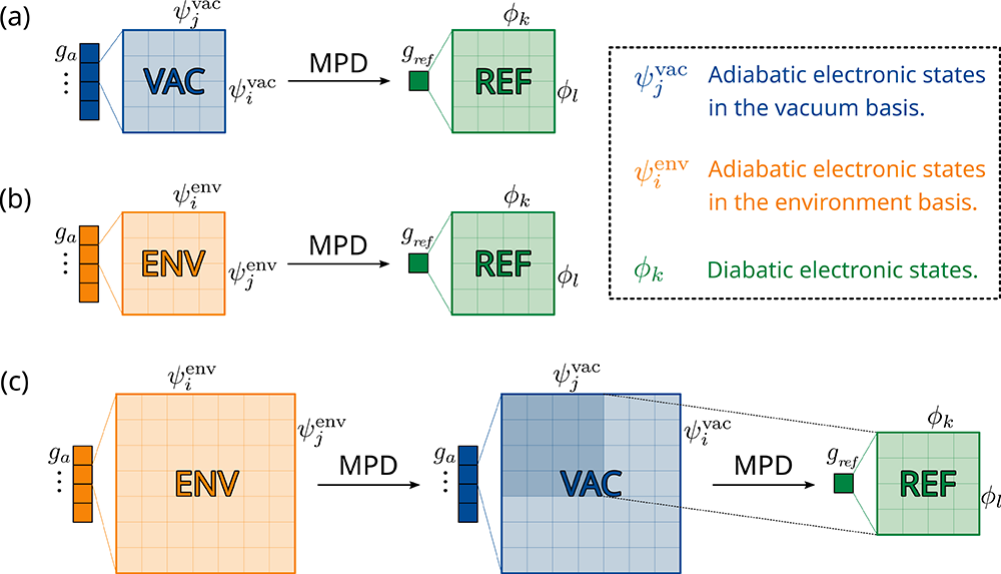
Schematic representation of the diabatization strategies adopted in this work. (a) Diabatization of electronic states in vacuum. (b) Diabatization of the electronic states in environment in one step. (c) Diabatization of environment electronic states in two steps: first the states are transformed in a “vacuum-like” representation using a large number of states, then the second step is done as in (a) using a smaller set of states.

### 2.2 Treatment of the Environment in Obtaining Adiabatic-to-Diabatic Transformations

Understanding the effect of the environment on the electronic states of xanthophylls is fundamental to studying their role in biological systems. The environment can also enhance mixing between lower and higher energy states, which can make the 4-state model (Figure 2b) less reliable in the environment than in the vacuum case (see Section S10). For this reason, we address the environment case with a slightly modified approach with respect to the vacuum case, using a 2-step procedure to obtain diabatic states, as we illustrate in Figure 2c.

We account for the effect of the environment in our calculations using an electrostatic embedding QM/MM approach. The adiabatic states in the presence of the environment are assumed to be obtainable via a transformation of the corresponding vacuum states (eq 11):

$$\left|\psi_{i}^{\mathrm{env}}\rangle =\sum_{j=1}^{n_{s}}T_{ij}|\psi_{j}^{\mathrm{vac}}\right\rangle$$ 11

and that the same holds for the properties computed from those states. This means that, with a similar approach used for obtaining the ATD transformation in vacuum, we can find a unitary transformation that rotates the environment states to resemble the vacuum states as much as possible. This step has the purpose of removing the mixing induced by the environment, and yields an intermediate “vacuum-like” representation rather than a fully diabatic basis. To this end, we use as a reference a calculation at the same QM geometry without the environmental charges. In the second step, we obtain the ATD transformation for the first 4 states (Figure 2), using the ground state equilibrium geometry properties as a unique reference.

### 2.3 Computational Details

Ground-state geometry optimizations in the gas phase were performed for all five xanthophylls at the DFT B3LYP/6–31G­(d) level of theory. Starting from the optimized geometries, ground-state relaxed scans along the BLA coordinate were carried out in the gas phase by performing constrained geometry optimizations at fixed BLA values, using Generalized Internal Coordinates (GIC) as implemented in the Gaussian package.^77^ A step size of 0.025 Å was employed. All geometry optimizations were carried out using the Gaussian 16 software package.^77^

Vertical excitation energies were computed as single-point calculations at the Franck–Condon geometry and at each structure along the BLA scans, using the methods described below.

FOMO–CI calculations^65^ were performed using a determinant space comprising all single and double excitations within the selected active orbital space (CISD). This reduced determinant space proved to be fast and effective in previous works on lutein^16,21,68–70^ and canthaxanthin.^23,71^ A modified AM1 Hamiltonian, reparameterized for lutein in ref 16, was adopted. An active space of 6 electrons in 9 orbitals was employed for all xanthophylls (R-AM1/FOMO–CISD­(6,9)). This active space was previously adopted for lutein^69,70^ and was selected in this work as it provides a balanced description for all the investigated carotenoids. The Gaussian energy width for the floating occupation of molecular orbitals was set to 0.1 Hartree. All calculations were carried out using the open-source MOPAC-PI software.^78^

DFT/MRCI calculations^53,54^ were carried out using the parallel version of the DFT/MRCI code^55^ with the R2022 Hamiltonian.^58^ The underlying DFT reference employed the BH&HLYP functional together with the def2-SVP basis set. The calculations were performed with the TURBOMOLE software package.^79^

MRSF-TDDFT calculations^49,51,52^ were carried out using the BH&HLYP functional and several members of the “double tuning” Coulomb-attenuated method (DTCAM) family, including DTCAM-AEE and DTCAM-VEE,^50^ as well as DTCAM-VAEE, DTCAM-XI, and DTCAM-XIV.^51^ The recently developed DTCAM-STG^80^ functional was also tested. All calculations employed the def2-SVP basis set. The calculations were performed using the Open Quantum Platform (OpenQP) package.^49,81–83^

For all methods, the terminal hexose sugar and hydrocarbon tail of salinixanthin were excluded from excited-state calculations to reduce the computational cost (see Figure S2).

QM/MM calculations were performed for canthaxanthin (Can) within the OCP protein environment. The Can chromophore was treated as the QM subsystem, while the rest of the protein was described at the MM level using the AMBER ff14SB force field.^84^ Electrostatic embedding was employed to describe QM-MM interactions. Relaxed scans along the BLA coordinate were carried out using the ONIOM scheme^85^ at the B3LYP/6–31G­(d) level for the QM region, consistent with the gas phase calculations. During these optimizations, the MM regions were kept frozen. QM/MM excitation energies were computed at the FOMO–CISD level by using the same parameters specified above for the isolated chromophores. These calculations were performed with MOPAC-PI^78^ interfaced with TINKER 6.3.^86^

## 3 Results and Discussion

### 3.1 Excitation Energies

We start our analysis by comparing the vertical excitation energies of xanthophylls computed with different methods. To this end, we focus on excited state properties of five representative systems: violaxanthin (Vio), lutein (Lut), zeaxanthin (Zea), canthaxanthin (Can), and salinixanthin (Sal), whose chemical structures are shown in Figure 1a.

All excited-state calculations were performed on ground-state geometries optimized at the DFT B3LYP/6–31G­(d) level of theory. The choice of the B3LYP^87^ functional for geometry optimization is motivated by previous work,^31^ which demonstrated that for extended π-conjugated systems, including xanthophylls, B3LYP-optimized geometries closely reproduce structural parameters obtained at the second-order Mo̷ller-Plesset (MP2) level of theory. In turn, MP2 agrees with third-order coupled cluster (CC3) calculations for smaller reference systems.^31^ We further assessed the BLA of Can computed at the ground state minima optimized using MP2 and DFT with different functionals, as shown in Section S2 of the Supporting Information (Figure S3). This analysis confirms that DFT/B3LYP shows significantly better agreement with MP2 and double-hybrid references, whereas CAM-B3LYP^88^ markedly overestimates the BLA.

Figure 3 reports the vertical excitation energies for the first three excited states, along with the corresponding transition dipole moments (TDM) for each studied xanthophyll (see Table S1). The excited states are labeled as A_*g*_^–^, B_*u*_^+^, B_*u*_^–^ based on visual inspection of the transition density and its (pseudo)­symmetry (we show the transition densities for all xanthophylls in Figures S4–S8). With this assignment, B_*u*_^+^ is the state with the larger transition dipole moment, A_*g*_^–^ is the dark state of lowest energy, and B_*u*_^–^ is the higher-energy dark state.

**Figure 3:**
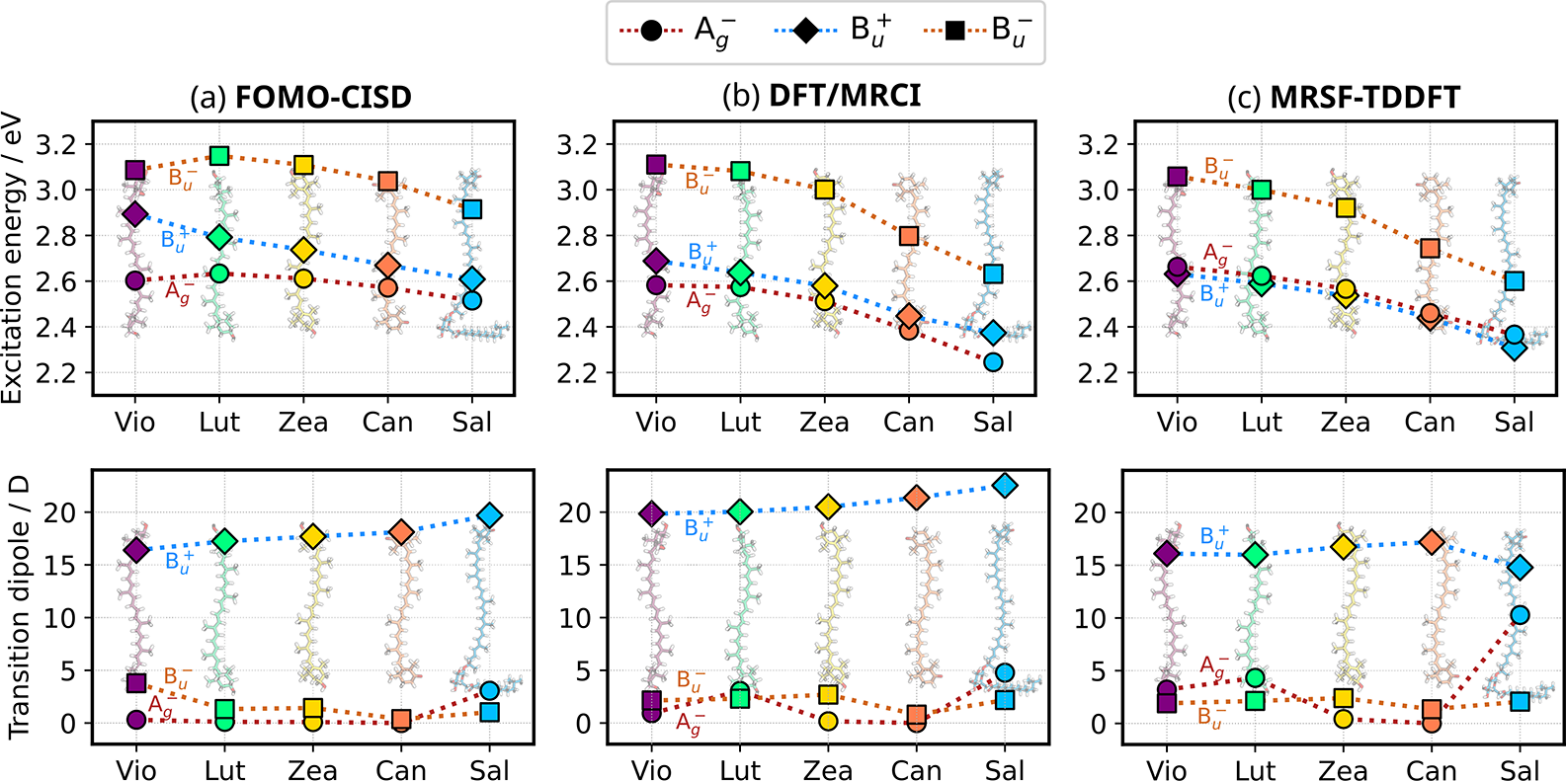
Comparison of vertical excitation energies (top panels) and transition dipole moments (bottom panels) for the three low-lying excited states of the xanthophyll set, computed on B3LYP/6–31G­(d) ground-state geometries with (a) FOMO–CISD­(6,9), (b) DFT/MRCI (BH&HLYP/def2-SVP), and (c) MRSF-TDDFT (DTCAM-XIV/def2-SVP). Excited states are denoted as A_*g*_^–^ (circles), B_*u*_^+^ (diamonds), and B_*u*_^–^ (squares).

All of the methods consistently reproduce the same qualitative trend in excitation energies for a given state among the different xanthophylls, which follows the ordering Vio > Lut > Zea > Can > Sal (Figure 3, Table S1). For the B_*u*_^+^ state, this order corresponds to the one observed in experimental measurements (Table S2). The only deviation is represented by Vio at the FOMO–CISD level (Figure 3a), where the excitation energy of the dark states appears slightly underestimated with respect to the bright B_*u*_^+^ state. For FOMO–CISD calculations, the same (6,9) active space was adopted for all xanthophylls based on preliminary benchmarks performed on lutein.^69,70^ We note that Vio has a shorter conjugation length in comparison to the other carotenoids (Figure 1a), and the epoxidic groups partially extend the conjugation to the rings,^89^ affecting the excited states. Our results suggest a mismatch in the employed semiempirical parameters, which are optimized for Lut,^16^ or possibly in the active space employed in the FOMO–CI calculations, which would not be adequate for Vio. System-specific parametrization may thus be required to achieve a balanced description of the excited-state manifold across the full set of xanthophylls.

Overall, the vertical excitation energies computed at the FOMO–CISD and DFT/MRCI levels are in close agreement for all the investigated xanthophylls (Figure 3a,b, Table S1). This consistency supports, in principle, the strategy adopted in our recent studies, which employs the semiempirical FOMO–CISD approach to describe the excited-state dynamics of xanthophylls in different protein environments,^16,21,23,69–71^ as a computationally efficient alternative to the more expensive DFT/MRCI, which has been largely employed to describe carotenoids and other polyenes.^45,46,54,56,58^ Comparison with experimental excitation energies (Table S2) also shows that the latest R2022 Hamiltonian^58^ has strongly improved the performance of this method on xanthophylls over the previous parametrizations.^45,46^

Let us now evaluate the performance of MRSF-TDDFT against our DFT/MRCI results, which are used here as reference data (see Section S4). We first consider the BH&HLYP/def2-SVP setup, which is commonly adopted for MRSF-TDDFT calculations.^52,90–92^ Consistent with previous benchmark studies on small polyenes such as butadiene^51^ and, more recently, lutein,^92^ this approach systematically overestimates excitation energies for all the xanthophylls considered here. More critically, this setup incorrectly predicts an inversion of the low-lying 2A_*g*_^–^ and 1B_*u*_^+^ states relative to the DFT/MRCI reference, as shown in Figure S10. Remarkably, the 1B_*u*_^+^ state remains ∼0.4 eV below 2A_*g*_^–^ also at the MRSF-TDDFT/BH&HLYP optimized geometry (Table S3). Because the energetic ordering of these states governs the photophysics of these molecules, such an inversion represents a qualitative failure.

Among the alternative DTCAM functionals explored, DTCAM-XIV provides the most consistent agreement with DFT/MRCI (Figure S10). As shown in Figure 3c, it reproduces both the qualitative trends across the xanthophyll series and, to a large extent, the quantitative excitation energies obtained from DFT/MRCI and FOMO–CISD calculations. We note that the DTCAM-XIV functional still predicts the 2A_*g*_^–^/1B_*u*_^+^ inversion also observed with BH&HLYP for all studied xanthophylls (see Figure S13). Nevertheless, in this case, the predicted energy gap between the two states is very small, and therefore, even slight variations along the BLA coordinate could readily reverse their ordering. The improved performance is consistent with the design principles underlying DTCAM-XIV, which was optimized to describe valence excitation energies and ionization potentials simultaneously through the combined application of double tuning and valence attenuation.^51,52^ These features appear to mitigate the systematic overestimation observed with BH&HLYP and to better balance the exchange-correlation treatment in xanthophylls.

The transition dipole values for Salinixanthin in Figure 3 merit further discussion. In particular, the transition dipole moment for the 2A_*g*_^–^ state is significantly larger than for the other xanthophylls with all methods, and much larger with MRSF-TDDFT. Analysis of the transition densities (Figure S8) reveals that the 2A_*g*_^–^ transition is partially delocalized on the carbonyl moiety, which enhances the asymmetry of the state. This effect is amplified with MRSF-TDDFT to the point that the transition densities of 2A_*g*_^–^ and 1B_*u*_^+^ are almost identical. This suggests a strong mixing of 2A_*g*_^–^ and 1B_*u*_^+^ at the DFT B3LYP/6–31G­(d) geometry. We report in Figure S9 the MRSF-TDDFT transition densities computed at the B3LYP minimum and at the MRSF-TDDFT minimum. At the latter geometry, it is easy to distinguish the two transition densities, which means that the two states are much less mixed. Figure S12 demonstrates that the B3LYP minimum is almost at the crossing point between 2A_*g*_^–^ and 1B_*u*_^+^, which explains the high degree of mixing.

In the following, the term MRSF-TDDFT refers specifically to calculations performed using the DTCAM-XIV functional unless otherwise stated. Despite the encouraging agreement observed for vertical excitation energies at the Franck–Condon point, additional validation is required to assess the robustness of MRSF-TDDFT/DTCAM-XIV for describing the excited-state landscape of xanthophylls more broadly (see below).

#### 3.1.1 Potential Energies across the Bond Length Alternation

We now move to evaluating how each method describes the PECs of these xanthophylls. One of the most relevant geometric coordinates in conjugated polyenes is the bond length alternation (BLA, definition in Figure 4). This coordinate provides the largest changes in excitation energies and is therefore a primary coordinate in the excited-state dynamics. Scanning along this coordinate allows us to investigate the electronic structure beyond the Franck–Condon point and to identify regions where electronic states cross or exchange their character. Accordingly, we performed ground-state B3LYP/6–31G­(d) relaxed scans along the BLA coordinate for the set of xanthophylls (see Section 2.3). Vertical excitation energies were then calculated on the resulting geometries to construct the PECs of the ground state S_0_ and the three lowest singlet excited states (S_1_–S_3_) using each method. Within this framework, we evaluate the energetic ordering of the bright and dark states and the crossings that arise along the BLA pathway. The results for Lut are shown in Figure 4, while the five xanthophylls are compared in Figure S13.

**Figure 4:**
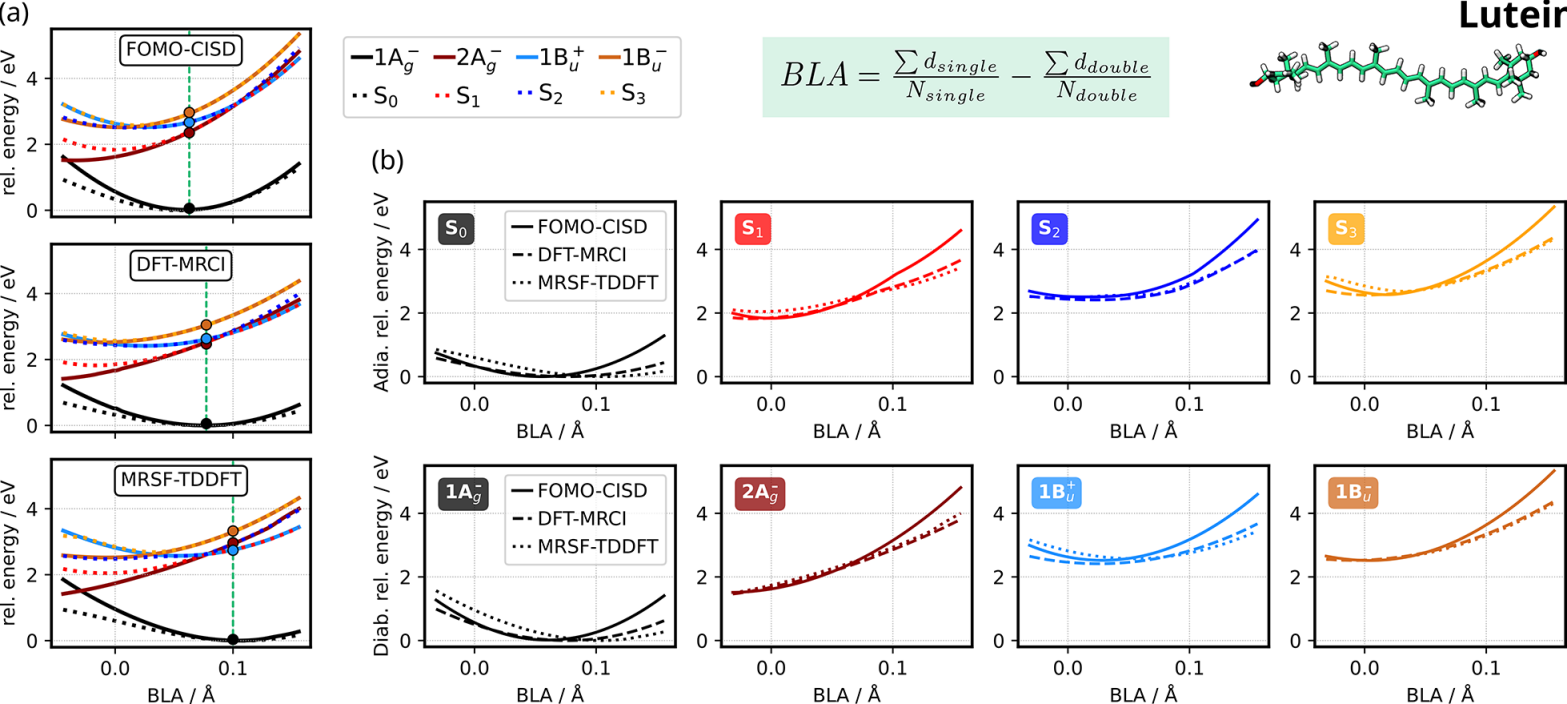
Comparison of electronic structure methods for the calculation of potential energy curves (PECs) of lutein along the bond-length alternation (BLA) coordinate. Panel (a) shows relaxed scans along the BLA coordinate for the ground (S_0_) and first three low-lying excited states (S_1_–S_3_), computed using FOMO–CISD, DFT/MRCI, and MRSF-TDDFT/DTCAM-XIV. Panels (b) present a direct comparison of the PECs obtained with the three methods for each electronic state, shown in the adiabatic (upper panel) and diabatic (lower panel) representations. The structure of lutein, as well as the definition of BLA are shown in the inset. The results for all the other carotenoids are reported in the Supporting Information (Figures S14–S18).

In order to understand the nature of the electronic character of the states along the PECs, we employed the MPD approach described in Section 2.1. The diabatic representation is essential to disentangling the complex excited-state pattern of xanthophylls, as it enables a continuous tracking of the electronic character of the states through the crossing regions. The MPD scheme can be consistently applied to all of the electronic-structure methods considered here, yielding a unified and physically meaningful description of the excited-state evolution along the BLA coordinate. Importantly, the procedure does not rely on system-specific assumptions, making it generally applicable and well-suited for comparative studies across different computational frameworks. Details on the validation of the diabatization are presented in Section S10.

For each computational method, the geometry corresponding to the minimum of the S_0_ PEC (vertical green dashed line in Figure 4a) was selected as a reference point for diabatization. As shown in Figure 4a, this procedure yields diabatic potentials (solid lines) that are shown together with the adiabatic PECs (dashed lines), enabling the assignment of the electronic character of each state and facilitating a more consistent comparison among the different computational methodologies. In fact, discrepancies between methods are expected to depend on the state character; therefore, errors may not be consistent when states are described in the adiabatic representation, whereas they are expected to be more systematic in the diabatic framework.

Figure 4a reveals qualitative trends that are common for all methods. First, with decreasing BLA, the covalent 2A_*g*_^–^ state drops in energy much more steeply than the two B_*u*_ states, and generates a strongly avoided crossing with the ground state at negative BLA. Second, the two B_*u*_ states show a crossing at lower BLA values than the Franck–Condon point. As such, the identity of the optically bright state evolves along the BLA coordinate through a sequence of state crossings involving the three lowest excited states, in agreement with previous studies on xanthophylls.^16,23,31,57,69,93^

More pronounced differences between the methods emerge when examining the detailed topology of the potential energy curves. A direct state-by-state comparison is shown in Figure 4b for both adiabatic (top) and diabatic (bottom) states. Surprisingly, in both adiabatic and diabatic representations, the largest discrepancy between methods is found in the ground-state PEC and in the position of its minimum. Here, DFT/MRCI and MRSF-TDDFT show the largest differences among them and with FOMO–CISD. The latter predicts a S_0_ minimum at BLA ≈ 0.056 Å, much smaller than the other methods (0.076–0.106 Å). Notably, the B3LYP optimized geometry of lutein corresponds to a BLA of 0.081 Å, which is closer to the DFT-based methods.

A comparison of the excited-state PECs reveals a remarkable overall agreement between DFT/MRCI and MRSF-TDDFT, in both the adiabatic and diabatic representations. The largest deviations between these two approaches occur in the negative BLA region, where MRSF-TDDFT has been shown to provide a less accurate description,^49,51^ a limitation that is confirmed here for xanthophylls. In contrast, at positive BLA values, relevant for the Franck–Condon region, the agreement between DFT/MRCI and MRSF-TDDFT is particularly good, supporting the reliability of MRSF-TDDFT in this physically relevant regime.

FOMO–CISD tends to exaggerate the curvature of both ground- and excited-state energy profiles as the system moves away from the Franck–Condon region, leading to a steeper increase in energy at large positive BLA values. This behavior is also reflected in the relative positioning of state crossings. In particular, the location of the 2A_*g*_^–^/1B_*u*_^+^ crossing differs among the methods: both FOMO–CISD and DFT/MRCI place this crossing at larger BLA values than the Franck–Condon point, resulting in S_2_ having predominant 1B_*u*_^+^ character in that region, whereas MRSF-TDDFT predicts a different ordering.

Finally, we analyze MRSF-TDDFT/BH&HLYP, since it is the most widely used setup in the literature. In the diabatic representation, the potential energy curves obtained from MRSF-TDDFT/BH&HLYP exhibit a curvature and overall shape that closely resemble those predicted by DFT/MRCI (see Figure S11). The main discrepancy instead lies in the absolute energies, which are systematically overestimated for the dark 2A_*g*_^–^ and 1B_*u*_^–^ states. This trend is clearly observed in the corresponding relaxed scan (Figure S11), while the bright 1B_*u*_^+^ state remains in good agreement with the reference DFT/MRCI. This affects the placement of the crossing regions in the adiabatic representation. In the following, we demonstrate that correcting the diabatic energies of the dark states is sufficient to recover the crossing points predicted by the other methods. To do so, we apply state-specific energy shifts in the diabatic representation, followed by rediagonalization to obtain corrected adiabatic PECs, as illustrated in Figure 5.

**Figure 5:**
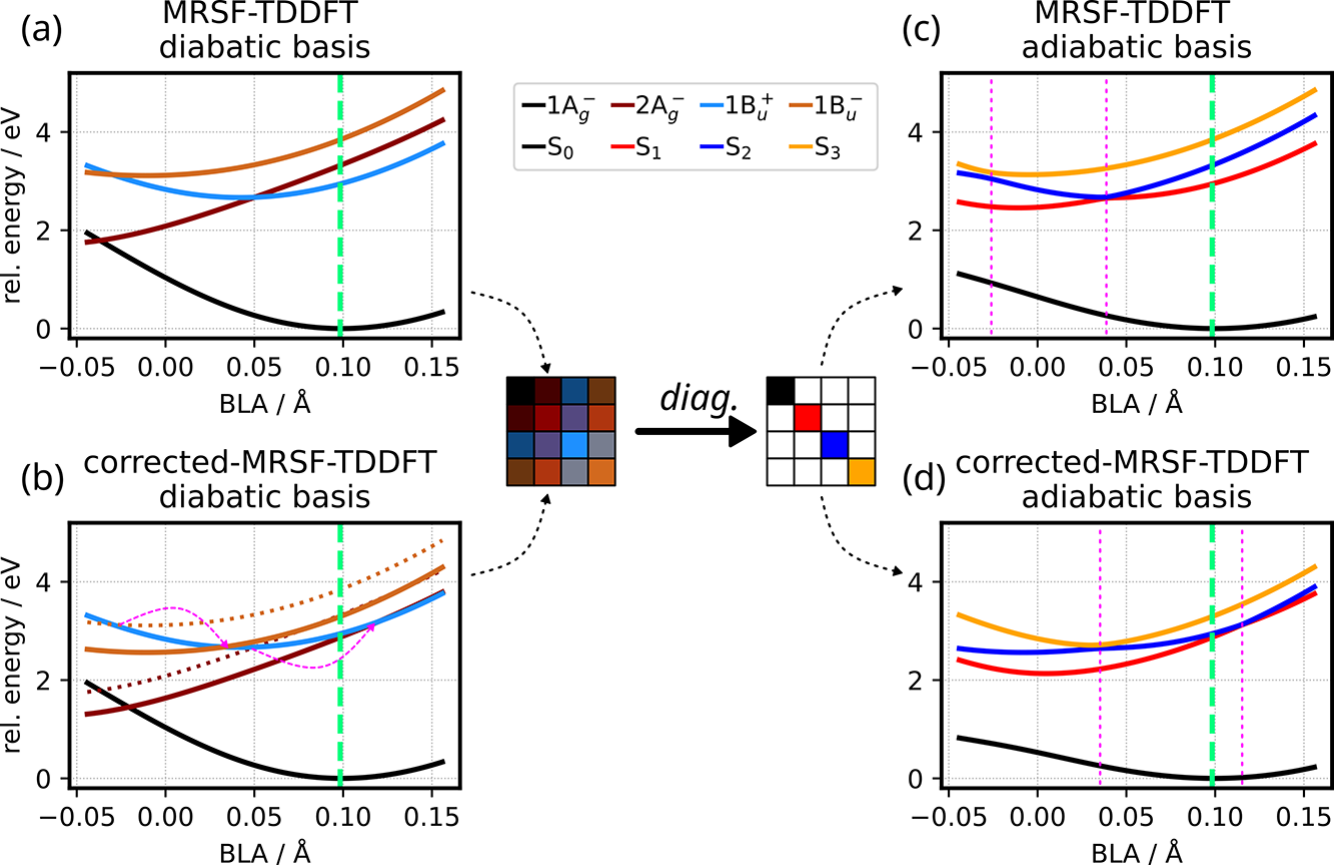
Diabatic and adiabatic energies computed with MRSF-TDDFT/BH&HLYP/def2-SVP on the B3LYP/6–31G­(d) relaxed scan over the BLA. (a) Diabatic energies obtained with 4-state MPD from states computed with MRSF-TDDFT/BH&HLYP/def2-SVP. (b) Original adiabatic energies. The position of the two crossing regions are highlighted with purple vertical lines. (c) Diabatic energies in which the 2A_*g*_^–^ and 1B_*u*_^–^ states are corrected targeting DFT/MRCI, subtracting a constant to the whole range. The correction factors are 0.45 eV for 2A_*g*_^–^ and 0.55 eV for 1B_*u*_^–^. (d) Adiabatic energies obtained after diagonalizing the corrected diabatic Hamiltonian.

In particular, we shift the original diabatic energies (Figure 5a) of two dark states, 2A_*g*_^–^ and 1B_*u*_^–^, by –0.45 eV and –0.55 eV, respectively. These vertical energy corrections induce substantial shifts in the intersections of the dark states with the bright state (Figure 5b). This effect becomes evident when comparing the original adiabatic PECs (Figure 5c) with the corrected adiabatic PECs obtained after diagonalization (Figure 5d). Notably, the latter displays a very similar behavior to that predicted by DFT/MRCI calculations. From this analysis, we can conclude that the main discrepancy between MRSF-TDDFT/BH&HLYP and the other methods arises from an overestimation of the energies of the covalent excited states.

Overall, these results demonstrate that all three methods reproduce the established qualitative photophysical picture of xanthophylls, barring the systematic overestimation of covalent states by MRSF-TDDFT with the BH&HLYP functional. Given the well-documented performance of DFT/MRCI and the close agreement observed here, MRSF-TDDFT with the DTCAM-XIV functional proves to be a promising alternative for the study of low-lying excited states of xanthophylls. The FOMO–CISD method, on the other hand, represents the best compromise between accuracy and computational cost. This method, with an adequate reparametrization of the semiempirical Hamiltonian,^16^ is able to closely reproduce the excited-state properties of DFT/MRCI. Although FOMO–CISD exaggerates the curvature of all PECs, this does not alter the positions of crossing points. This explains the success of semiempirical FOMO–CISD in describing the ultrafast excited-state dynamics of Lut^16,21,69^ and Can^23,71^ in a protein environment. A different reparameterization, specifically targeting out-of-equilibrium geometries, could address the remaining issues.

#### 3.1.2 Comparison with Ab Initio Multireference Calculations

The results above show that the three cost-effective approaches (FOMO–CISD, DFT/MRCI, and MRSF-TDDFT) predict very similar potential energy curves for both the adiabatic and the diabatic states. We now assess their reliability by comparison with a higher-level ab initio reference (see Section S9), which is expected to provide a more accurate description of the electronic structure. As reference data, we use the multireference perturbation theory excitation energies reported in ref 15 for Lut, Zea, and Vio. In that study, vertical excitation energies were computed at the DSRG-MRPT2 level on ground-state DFT CAM-B3LYP/cc-pVDZ geometries. To ensure a consistent comparison, we performed our calculations on the same geometries, although CAM-B3LYP clearly overestimates the BLA (see above).

Table 1a reports the vertical excitation energies of the first two excited states (S_1_ and S_2_) at the ground-state minimum computed on the CAM-B3LYP/cc-pVDZ geometries, together with the corresponding DSRG-MRPT2 reference values. The state with the largest transition dipole moment (highlighted in bold) is not always S_2_, depending on the geometry. We report also the S_1_–S_2_ energy gap, which is taken to be positive regardless of the state ordering.

**Table 1:** Vertical Excitation Energies (in eV) of the First Two Excited States (S_1_, S_2_) and Their Energy Gap (Δ) Computed at the Ground-State Minimum Geometries Obtained with (a) CAM-B3LYP/cc-pVDZ (Ref 15) and (b) B3LYP/6-31G­(d) (This Work)^a^

(a) CAM-B3LYP geometry												
	**DSRG-MRPT2** ^15^			**FOMO–CISD**			DFT/MRCI			**MRSF-TDDFT**		
Car	S_1_	S_2_	Δ	S_1_	S_2_	Δ	S_1_	S_2_	Δ	S_1_	S_2_	Δ
Vio	2.99	**3.01**	0.02	2.94	**3.06**	0.12	**2.86**	2.91	0.05	**2.82**	3.05	0.23
Lut	2.95	**2.97**	0.02	**2.97**	3.04	0.07	**2.82**	2.91	0.09	**2.80**	3.03	0.23
Zea	**2.90**	2.92	0.02	**2.93**	2.98	0.05	**2.78**	2.87	0.09	**2.76**	3.00	0.24

(b) B3LYP geometry									
	**FOMO–CISD**			DFT/MRCI			**MRSF-TDDFT**		
Car	S_1_	S_2_	Δ	S_1_	S_2_	Δ	S_1_	S_2_	Δ
Vio	2.60	**2.89**	0.29	2.58	**2.69**	0.11	**2.63**	2.66	0.03
Lut	2.63	**2.79**	0.16	2.57	**2.64**	0.07	**2.59**	2.62	0.03
Zea	2.61	**2.74**	0.13	2.51	**2.58**	0.07	**2.54**	2.57	0.03

a The state with the largest transition dipole moment is shown in bold. FOMO–CISD, DFT/MRCI, and MRSF-TDDFT results are from this work; DSRG-MRPT2 values^15^ are used as reference

In the DSRG-MRPT2 reference data, the bright state corresponds to S_2_ for Vio and Lut, and to S_1_ for Zea, indicating that for Zea the S_1_/S_2_ crossing lies to the left of the ground-state minimum. However, the two states are almost degenerate (the S_1_–S_2_ gap is ≈0.02 eV) for all three xanthophylls. The other methods all yield somewhat larger energy gaps, and predict the bright state to be S_1_. The only exception is Vio at the FOMO–CISD level.

To put these results in perspective, we note that the estimated error of the DSRG-MRPT2 reference is in the order of 1000 cm^–1^ (0.12 eV) and depends on the state.^15^ The S_1_–S_2_ gaps predicted by FOMO–CISD and DFT/MRCI are within 0.12 eV, and the discrepancy between these methods and the reference is roughly of the same magnitude. Hence, these methods prove to be in good agreement with the reference within its expected accuracy. A larger discrepancy is found for MRSF-TDDFT, which places the dark state about 0.2 eV above the bright state at this geometry. Therefore, even with the DTCAM-XIV functional, MRSF-TDDFT slightly overestimates the energy of the 2A_*g*_^–^ state.

Overall, FOMO–CISD shows a very good agreement with reference data, even for molecules outside the initial parametrization. On the other hand, the trend in the 2A_*g*_^–^ energies is not well reproduced, since the 2A_*g*_^–^ energy of Vio is well below that of Lut. This discrepancy may again be attributed to the epoxidic moiety, as discussed above. DFT/MRCI seems to slightly underestimate both states, and in particular the bright state, placing it almost 0.1 eV below the dark state. As discussed above, however, the discrepancy is within the estimated error of the reference. Overall, the R2022 Hamiltonian significantly improves the description of xanthophyll excitation energies compared to the earlier parametrization tested in ref 15, which markedly underestimated the vertical excitation energies.

At the B3LYP-optimized geometries (Table 1b), the state ordering changes: only MRSF-TDDFT predicts S_1_ as the bright state, although the two states are almost degenerate. The smaller bond-length alternation of this geometry (BLA ∼0.08 vs ∼0.10 Å for CAM-B3LYP) shifts the dark state lower than the bright one, as the former has a steeper dependence on the BLA (see Figure 4). As such, at this geometry we recover the expected ordering in the Franck–Condon region, where the B_*u*_^+^ state is generally assigned to S_2_.

Taking these results together, we have shown that (i) the methods tested here agree with the MRPT reference, or at most overestimate the energy of the 2A_*g*_^–^ state, and (ii) at the B3LYP minimum, the 2A_*g*_^–^ is lower in energy or very close to the 1B_*u*_^+^ state. As the B3LYP geometry appears to be a better estimation of the ground-state minimum, we conclude that at the Franck–Condon point, the excited states are most likely in the 2A_*g*_^–^ < 1B_*u*_^+^ order for all the considered carotenoids. MRSF-TDDFT predicts the opposite order because it overestimates the 2A_*g*_^–^ energy by about 0.2 eV.

As a complementary analysis, in Section S9, we evaluate the performance of the investigated methods in computing the adiabatic energies of the 2A_*g*_^–^ and 1B_*u*_^+^ states (Table S4). While the agreement is less satisfactory, it is important to note that adiabatic energies depend on the methods employed to optimize both ground- and excited-state geometries, which limits the usefulness of this analysis for assessing the accuracy of the excited-state methods considered here.

### 3.2 Excited-State Properties

In this section, we examine the excited-state properties of the xanthophylls considered in this work, focusing on permanent and transition dipole moments as complementary probes of electronic structure. While transition dipole moments provide information on state symmetry and optical activity, state dipole moments reflect the spatial distribution of electronic charge and are therefore sensitive to charge-transfer character. Understanding how these quantities evolve with molecular symmetry in xanthophylls is essential, as electronic-state coupling plays a central role in determining their biological function. For instance, in nonphotochemical quenching (NPQ), efficient excitation-energy dissipation requires mixing between the S_1_/2A_*g*_^–^ and S_2_/1B_*u*_^+^ states, enabling the S_1_ state to act as an effective excitation-energy acceptor.^5,6,34,70,94–97^

State mixing arises from both intrinsic molecular features and external perturbations. While excited states in polyenes are typically classified within the C_2*h*_ point group, this description is strictly valid only for perfectly symmetric geometries. Under ideal C_2*h*_ symmetry, coupling between 2A_*g*_^–^ and 1B_*u*_^+^ states is symmetry-forbidden; mixing can therefore occur only upon symmetry breaking, either due to structural distortions that induce asymmetry or environmental effects.

#### 3.2.1 Effect of the Chemical Structure

We first compare gas-phase excited-state properties across the different xanthophylls. Lutein is selected as a representative asymmetric xanthophyll, while canthaxanthin serves as a symmetric reference. As shown in Figure 6, Lut is asymmetric because one terminal 3-hydroxyl ring contains an endocyclic double bond, whereas the other is connected through an sp^3^ carbon and is therefore not conjugated. In contrast, Can possesses two identical terminal rings with conjugated 4-keto-carbonyl groups.

**Figure 6:**
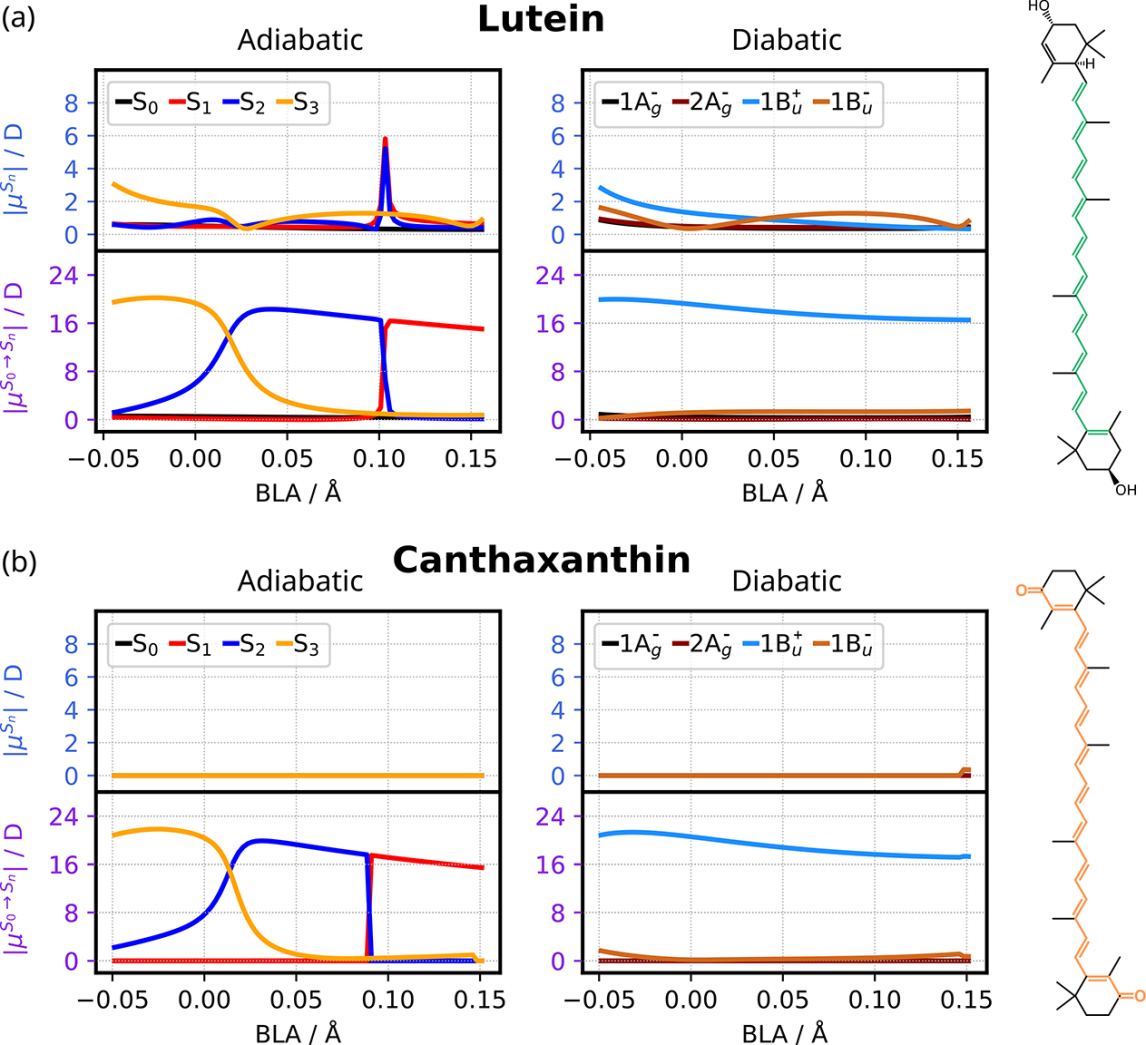
Permanent dipole moments and transition dipole moments of the first 4 electronic states of (a) lutein and (b) canthaxanthin computed at the FOMO–CISD level. The dipoles and energies of all carotenoids computed also with the other methods are reported in the Supporting Information (Figures S14–S18). The molecular structures of the two molecules are reported next to the plots. On the left panels, the states are in the adiabatic representation, on the right ones they are in the diabatic representation obtained with the MPD method.

We analyze the evolution of state dipole moments (μ^*S*_0_→*S*_*n*_^) and transition dipole moments (μ^*S*_*n*_^) along the BLA coordinate for Lut (Figure 6a) and Can (Figure 6b), in both adiabatic and diabatic representations. As observed, the transition dipole moments primarily identify the optically bright 1B_*u*_^+^ state, which is characterized by the largest oscillator strength. This state changes its adiabatic labeling from S_3_ to S_2_ and then to S_1_, along the BLA coordinate, clearly revealing two avoided crossings.

In contrast, the state dipole moments provide direct insight into the charge redistribution due to molecular distortion. In the ground state, Lut exhibits a nearly vanishing dipole moment, reflecting an almost symmetric charge distribution. However, near the S_1_/S_2_ crossing, pronounced peaks appear in the dipole moments of both of the involved states. The sudden emergence of a permanent dipole indicates the development of an internal charge-transfer (ICT) character,^22^ arising from strong coupling between the interacting states. Importantly, these dipole peaks disappear in the diabatic representation, where the states are uncoupled, suggesting that the ICT character is not intrinsic to a single diabatic state but emerges from its interaction.

On the other hand, Can does not exhibit analogous dipole enhancements. Although the transition dipole moments still identify the bright 1B_*u*_^+^ state, the state dipole moments remain zero because of symmetry. As the 2A_*g*_^–^/1B_*u*_^+^ coupling is symmetry-forbidden in vacuum, no charge-transfer character develops. These results suggest that ICT character originates from coupling between S_1_ and S_2_, that is, from mixing of 2A_*g*_^–^ and 1B_*u*_^+^ configurations, enabled by symmetry breaking.

#### 3.2.2 Effect of Environment Perturbation

While in a vacuum the ICT character appears only at the S_1_/S_2_ degeneracy point, environmental effects are expected to play a crucial role in stabilizing and enhancing this state. Electrostatic interactions and steric constraints imposed by the surrounding environment can perturb both the geometry and electronic structure, thereby modulating the extent of symmetry breaking and electronic-state mixing.

In a protein environment, steric interactions can distort the xanthophyll's structure, thereby breaking its symmetry. As a test case to illustrate the effect of the protein environment, we consider the Orange Carotenoid Protein (OCP, Figure 7a), a photoreceptor that enables photoprotection in cyanobacteria.^74,98–100^ This protein embeds a keto-carotenoid, here canthaxanthin, which acts both as a light-sensor for the activation of the protein complex and as the photoprotective agent in cyanobacteria's photosynthetic apparatus.^73,101^ Due to the constraints of the OCP binding pocket (in the inactive form, OCP^*O*^), the xanthophyll is significantly distorted relative to its vacuum equilibrium geometry (Figure 7b). In addition, the electrostatics of the protein pocket itself can polarize the xanthophyll, further breaking the symmetry of its electronic density.

**Figure 7:**
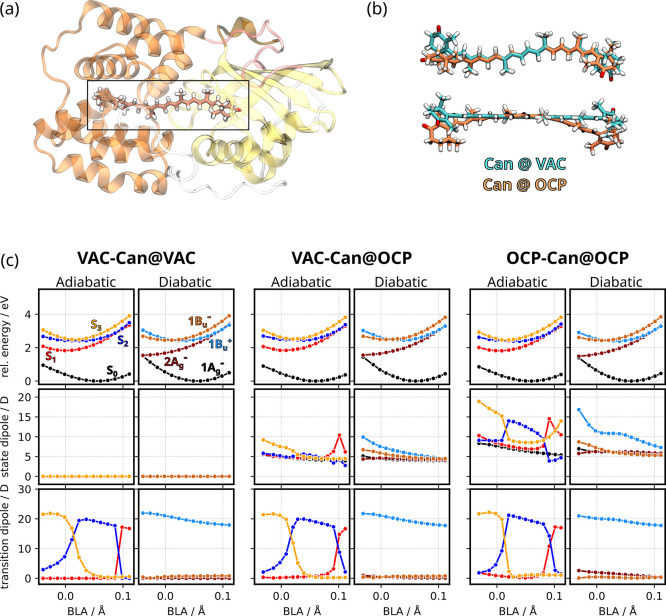
(a) Structure of OCP^*O*^ binding Can. (b) Structure of Can in vacuum (Can@VAC) and in OCP^*O*^ (Can@OCP). (c) Energies and (transition) dipole moments along the BLA of Can 1) optimized in vacuum, computed in vacuum (VAC-Can@VAC); 2) optimized in OCP, computed in vacuum (VAC-Can@OCP); 3) optimized in OCP, computed in OCP (OCP-Can@OCP). The calculations are performed at the FOMO–CISD level of theory. The results are reported both in the adiabatic and diabatic representation.

To disentangle the indirect and direct effects of the protein environment, we analyze the BLA relaxed scan of Can performed under three different conditions. VAC-Can@VAC denotes vacuum excited-state calculations on gas-phase optimized geometries (same as Figure S13), while VAC-Can@OCP refers to vacuum excited-state calculations on QM/MM geometries optimized within the OCP environment, capturing binding-pocket-induced distortions. Finally, OCP-Can@OCP corresponds to excited-state QM/MM calculations on QM/MM OCP-optimized geometries, thus accounting for both electrostatic and structural effects of the protein. These results are presented in Figure 7.

As shown by the S_0_–S_3_ PECs along the BLA (Figure 7c), the overall energy trends are preserved across all conditions, indicating that neither molecular distortion nor environmental electrostatics significantly affects the state energies. In contrast, the state dipole moments are completely different in the three cases. First of all, the dipole of all states, including the ground state, is zero only in the VAC-Can@VAC. The distortion of the molecule induces a constant dipole on the ground state, while the excited states are affected in different ways depending on the BLA. By comparing the two vacuum cases (i.e., VAC-Can@VAC and VAC-Can@OCP), it is evident how the symmetry breaking of Can, due to its interaction with the OCP environment, leads to the formation of a charged S_1_ state in correspondence to the S_1_/S_2_ intersection, as also observed for lutein in vacuum (Figure 6a). Consistently, the S_1_ dipole disappears in the diabatic representation.

When the OCP environment is included in the excited-state calculations, we observe an overall stronger increase of all the state dipoles. Specifically, the S_1_ peak in the crossing region has an increased value with respect to the gas-phase calculations, but there is also a strong effect on the other states (S_2_ and S_3_) mostly in the negative BLA region. In the diabatic representation, we clearly see that the affected state is 1B_*u*_^+^, which retains the dipole also in this representation.This dipole is most likely induced by the protein environment, consistent with the fact that the state most sensitive to the environmental polarity is the ionic one. The mixing between S_1_ and S_2_ can also be seen from the transition dipole moments. In the perfectly symmetric case (VAC-Can@VAC), the exchange of transition dipole moments between S_1_ and S_2_, as the states interchange their character, occurs sharply and abruptly (Figure 7c). In the other two cases, by contrast, the crossing occurs more gradually, and several points are observed where both S_1_ and S_2_ exhibit intermediate values of the transition dipole moment.

Overall, we showed how both the symmetry breaking and the direct effect of the environment contribute to S_1_/S_2_ state mixing. This effect can appear small for some observables, such as the state energy and the shape of the PECs, but it has a greater impact on other excited-state properties, such as the dipole and transition dipole moment. The diabatic representation here proposed is very useful to identify the origin of these property changes, as it removes the state mixing. In fact, it disentangles the S_1_ and S_2_ states, thus identifying the effects that derive from the mixing of the two states as they disappear in the diabatic representation.

## 4 Conclusions

In this work, we compared the DFT/MRCI, FOMO–CISD, and MRSF-TDDFT methods for the description of the low-lying excited states of representative xanthophylls. We focused on vertical excitation energies at the Franck–Condon point as well as potential energy curves along the bond-length alternation (BLA) coordinate. We also performed a consistent comparison of the electronic character across methods aided by a unified multiple-property diabatization scheme.

Our results show a remarkable consistency across methods, especially for DFT/MRCI and the semiempirical FOMO–CISD. They consistently predict the energetic ordering of the dark S_1_/2A_*g*_^–^ and bright S_2_/1B_*u*_^+^ states near the Franck–Condon point and yield closely matching vertical excitation energies across the xanthophyll series. Notably, FOMO–CISD provides a balanced description for all systems using a common active space and the original semiempirical parametrization for lutein,^16^ indicating good transferability across related carotenoids, although higher discrepancies are found for violaxanthin. Its main limitation arises away from the Franck–Condon region, where FOMO–CISD systematically overestimates the curvature of both ground- and excited-state potential energy surfaces. However, this does not substantially alter the position of crossing regions, which explains the success of FOMO–CISD for modeling ultrafast excited-state dynamics of carotenoids.^16,21,23,68,70,71^

Both DFT/MRCI and FOMO–CISD excitation energies are in close agreement (within 0.12 eV) with multireference perturbation theory reference values calculated at the CAM-B3LYP ground-state geometry. This places the uncertainty of both methods below 0.2 eV for the energies of the 2A_*g*_^–^ and 1B_*u*_^+^ states.

For MRSF-TDDFT, our results demonstrate a strong dependence on the DFT functional. The BH&HLYP functional systematically overestimates excitation energies of the dark states, leading to an incorrect state ordering. In contrast, the DTCAM-XIV functional yields excitation energies and potential energy profiles in close agreement with DFT/MRCI, particularly in the positive BLA region relevant to the Franck–Condon geometry. Deviations become more pronounced in the negative BLA region, where MRSF-TDDFT has been shown to be less reliable.^49,51^ The diabatic representation reveals that most of the discrepancy for BH&HLYP arises from systematic state-specific energy shifts. Correction strategies formulated in the diabatic representation may help to improve the potential energy surfaces.

The diabatization scheme employed here provides a consistent description of the state character and crossings across methods and systems, enabling a rigorous comparison of potential energy surfaces. Exploiting this scheme, we have shown how similar the descriptions of carotenoid excited states are by the various methods considered here. More generally, our work suggests that diabatization schemes can aid the analysis of electronic structures at different geometries and levels of theory.

All of the considered methods are able to predict excited-state properties of carotenoids consistently. In particular, they reliably capture the trend in transition dipole moments that reflect the exchange of character between adiabatic states. Our analysis of state mixing induced by the symmetry breaking and environmental effects provides a clear rationale for the emergence of intramolecular charge-transfer (ICT) character in the S_1_ and S_2_ states, specifically in regions where these states are more strongly coupled.

Our findings provide a consistent basis to interpret the photophysics of xanthophylls and establish practical guidelines for method selection in simulations of carotenoid photophysics.

## Supplementary Material



## Notes

Molecular structures of the xanthophylls investigated in this work; assessment of the impact of the geometry optimization method on the bond-length alternation (BLA), using canthaxanthin and lutein as a representative cases; visual representation of the transition densities and difference densities, computed with the FOMO–CISD, DFT/MRCI, and MRSF-TDDFT; benchmark of different DTCAM MRSF-TDDFT functionals in terms of excitation energies and BLA profiles along scans for the three lowest-lying excited states of the studied xanthophylls; results from the relaxed scans along the BLA coordinate for all investigated xanthophylls and methodologies; detailed analysis and validation of the MPD diabatization scheme employed; complete raw data of vertical energies at the B3LYP optimized geometry; MRSF-TDDFT vertical energies and transition dipoles at MRSF-TDDFT optimized geometries; compilation of available experimental data for the vertical excitation energy of the bright state in various solvents; and supporting analyses of vertical and adiabatic excitation energies computed using FOMO–CISD, DFT/MRCI, and MRSF-TDDFT (PDF)
