# Supplementary material for: Modeling Xanthophyll Excited States via Cost-Effective Quantum Chemistry methods and Property-Based Diabatization
Source: J Chem Theory Comput. 2026 Jun 20;22(13):6776–90. doi: 10.1021/acs.jctc.6c00637 (PMC13374009; doi:10.1021/acs.jctc.6c00637)
Supplement: Supplementary file 1 [file ct6c00637_si_001.pdf]

Supporting information for: Modeling Xanthophyll Excited States via Cost-Effective Quantum Chemistry methods and Property-Based Diabatization

Amanda Arcidiacono, Valentino Martini, Lorenzo Cupellini,* and Laura Pedraza-González*

Dipartimento di Chimica e Chimica Industriale, Università di Pisa, via G. Moruzzi 13, 56124 Pisa, Italy

E-mail: lorenzo.cupellini@unipi.it; laura.pedraza@unipi.it

Contents

S1 Structures of studied xanthophylls **S3**

S2 Effect of the geometry optimization method on BLA **S4**

S3 Visual representation of difference densities and transition densities **S5**

S4 Performance of MRSF-TDDFT functionals on vertical excitation energies and BLA profiles **S11**

S5 Comparison of BLA potential energy curves computed with different methods **S13**

S6 Raw data in vertical excitation energies **S19**

S7 Experimental data on the bright state ($1B_u^+$) **S20**

S8 Effect of the optimization on MRSF-TDDFT excitation energies **S20**

S9 Comparison with *ab initio* multireference calculations **S21**

S10 Diabatization analysis **S25**

S10.1 Choosing the diabatization reference S25

S10.2 Choosing the optimal number of states S27

References **S32**

S1 Structures of studied xanthophylls

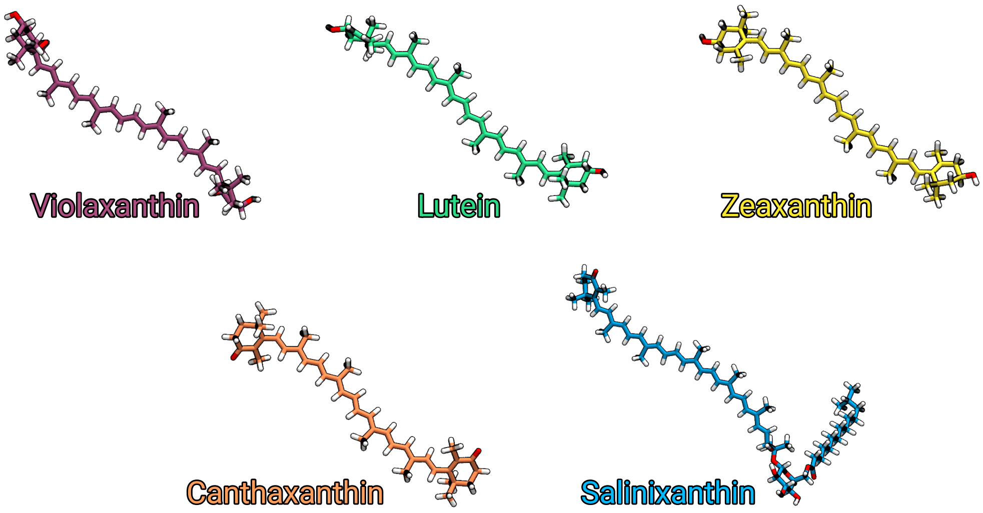

Figure S1: Molecular geometry of the carotenoids investigated in this work.

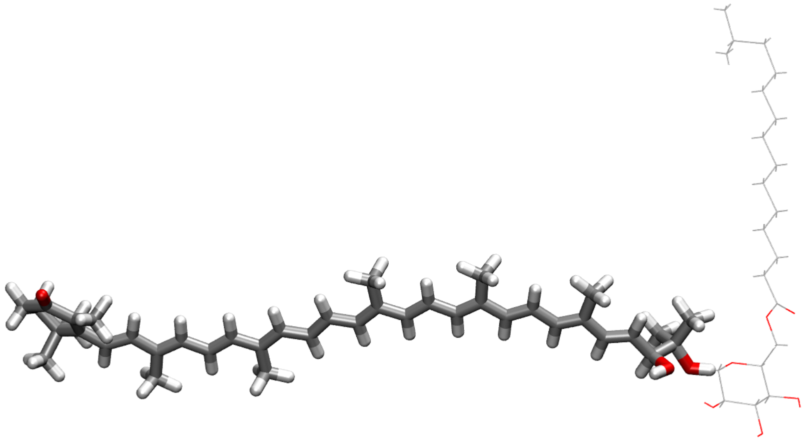

Figure S2: The model used for the excited state calculations of salinixanthin is represented in licorice, the part of the structure that was excluded from the calculation is represented in lines.

S2 Effect of the geometry optimization method on BLA

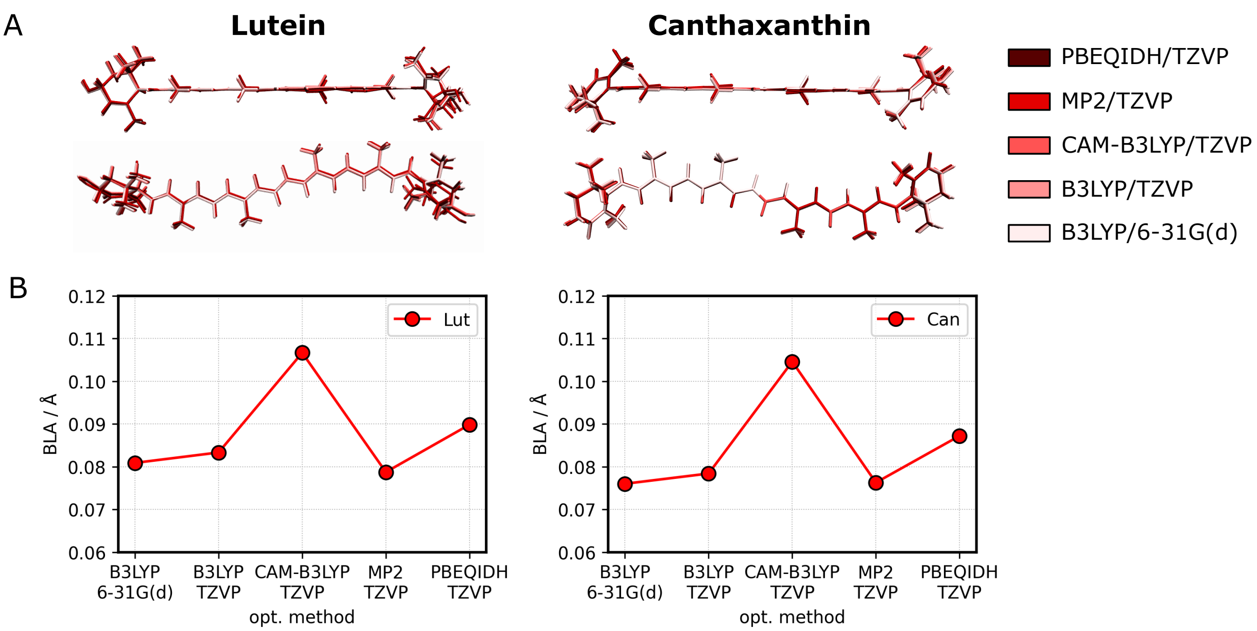

Figure S3: Optimizations of lutein and canthaxanthin at different levels of theory. (A) Superposition of optimized geometries; (B) Bond lenght alternation (BLA) of the different optimized geometries.

S3 Visual representation of difference densities and transition densities

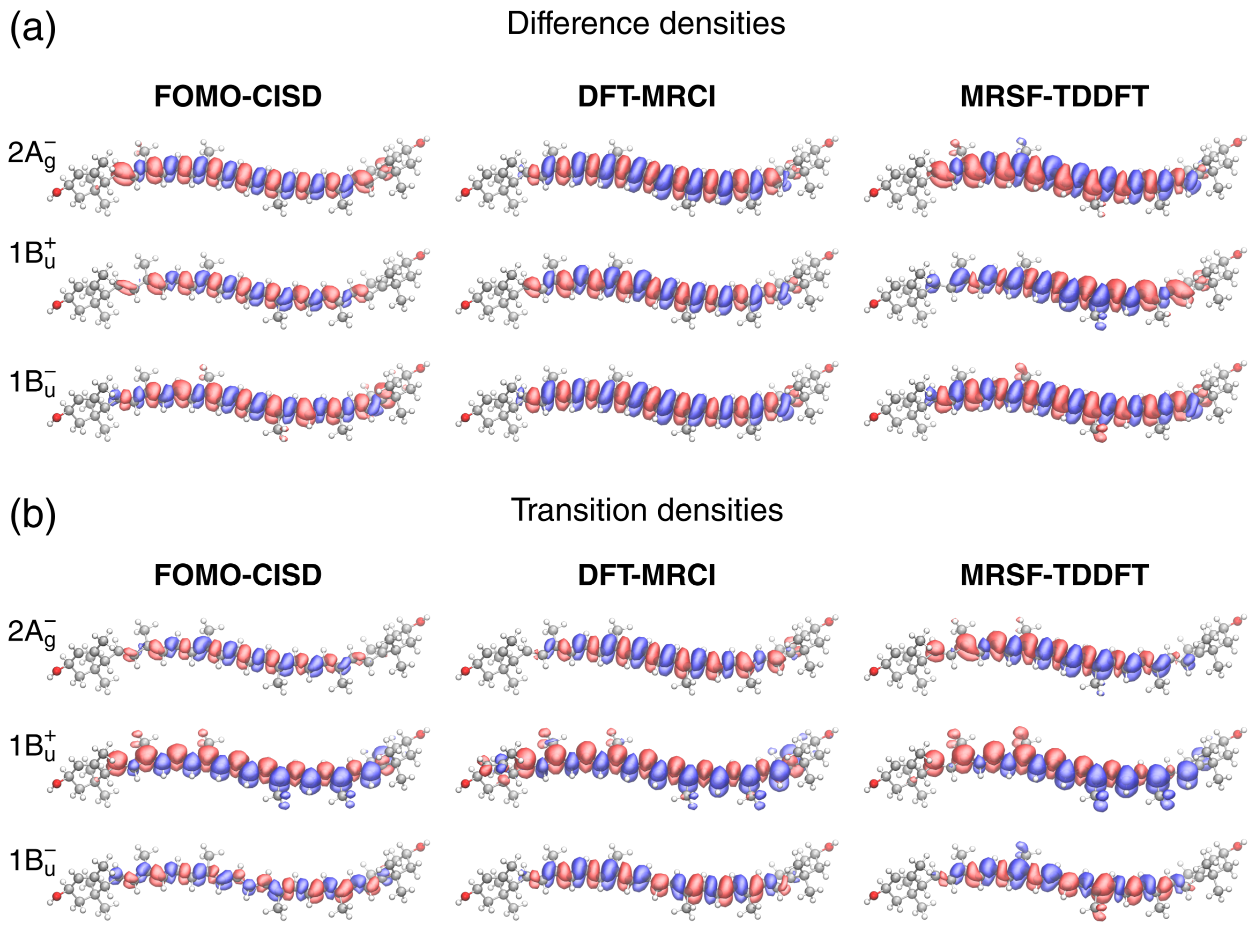

Figure S4: Excited-state characterization for **Lutein**. (a) Difference densities (ES − GS, blue/red for positive/negative isovalues) and (b) transition densities (GS-to-ES) calculated with different methods, at the DFT B3LYP/6-31G(d) ground-state geometry. Adiabatic excited states calculated with MRSF-TDDFT have been reordered to match the other methods. The pseudo-symmetry (A/B) and covalent character (+/−) of the states is evident from the transition densities. Note the partial mixing between 2A$_g^-$ and 1B$_u^+$ in the MRSF-TDDFT results.

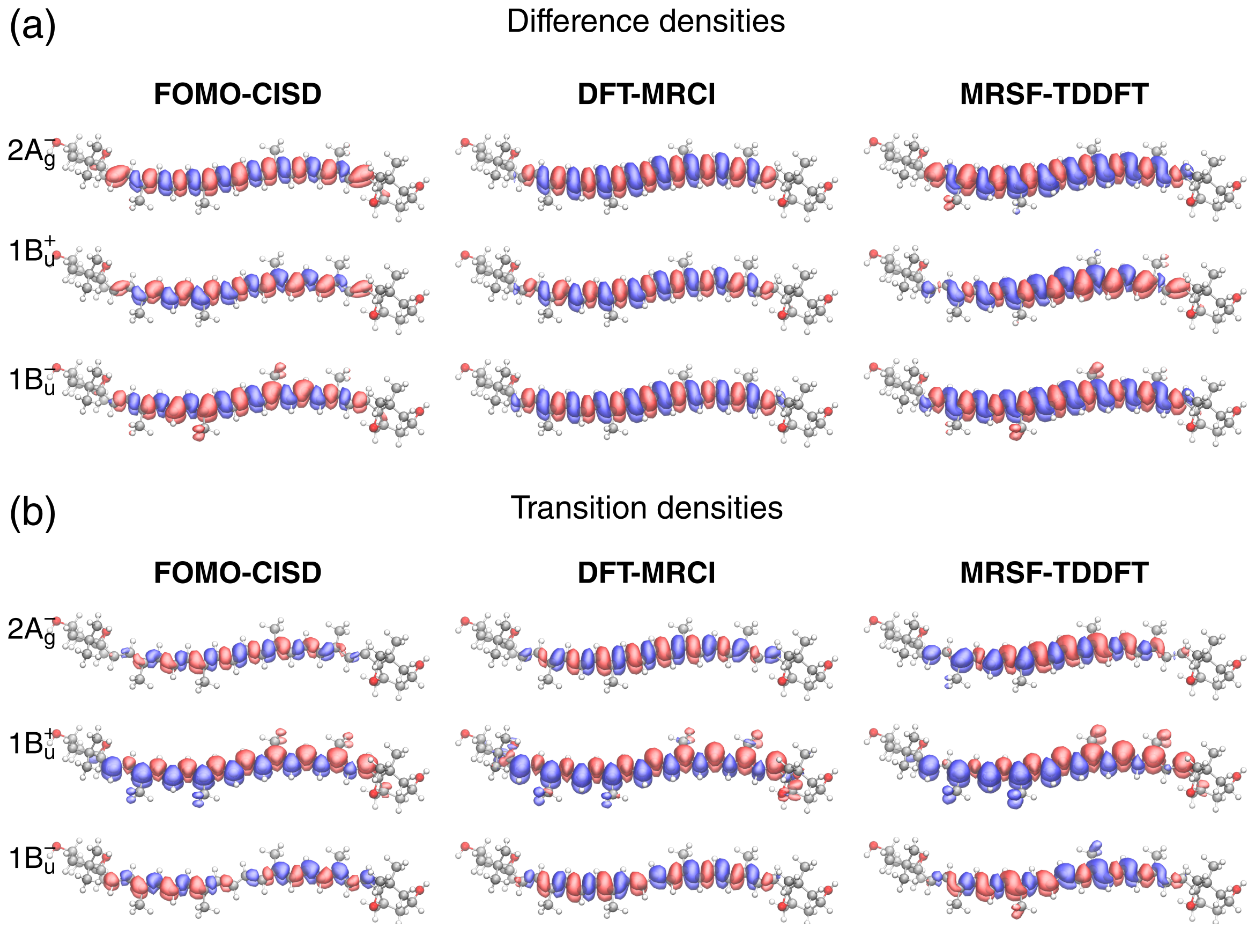

Figure S5: Excited-state characterization for **Violaxanthin**. (a) Difference densities (ES − GS) and (b) transition densities (GS-to-ES) calculated with different methods, at the DFT B3LYP/6-31G(d) ground-state geometry. Adiabatic excited states calculated with MRSF-TDDFT have been reordered to match the other methods. The symmetry (A/B) and covalent character (+/−) of the states is evident from the transition densities.

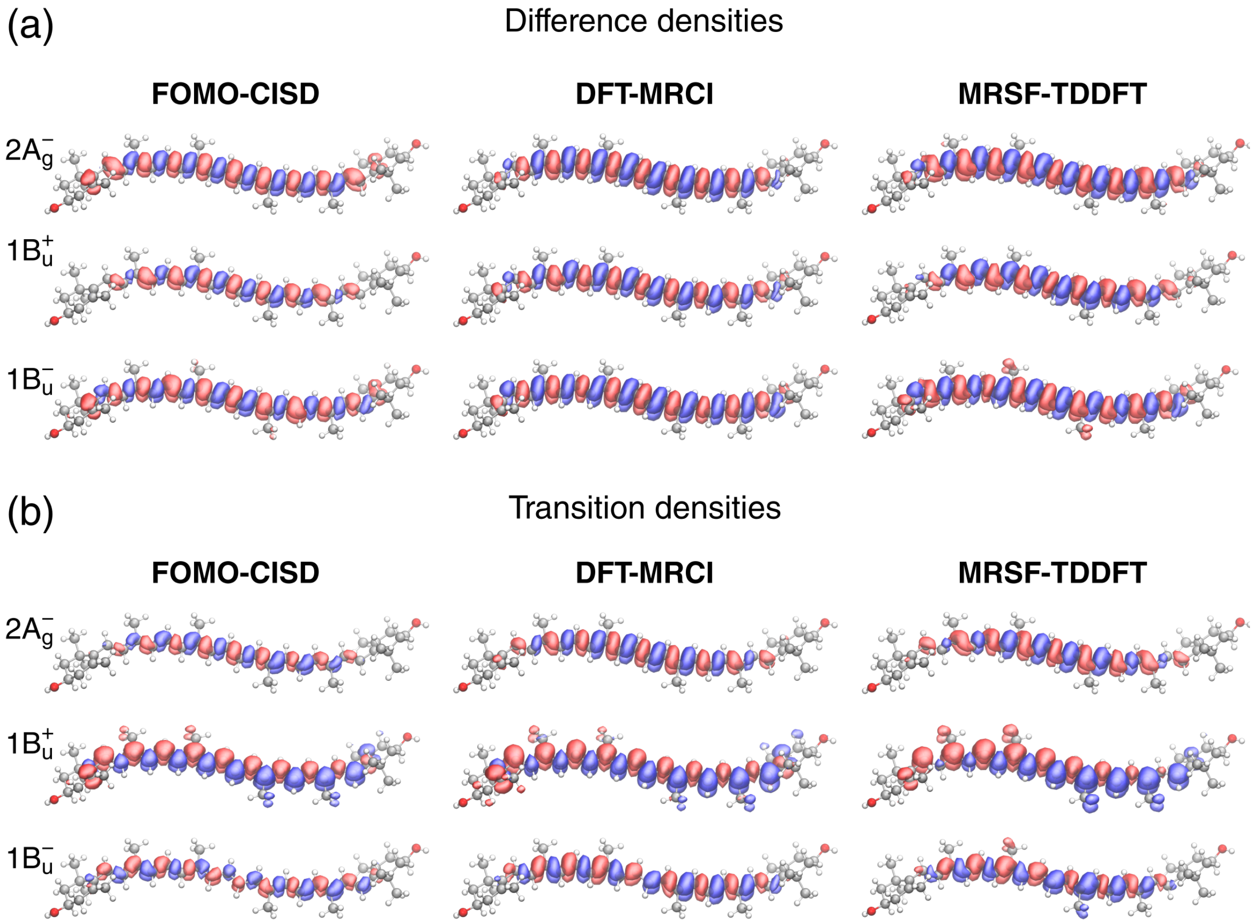

Figure S6: Excited-state characterization for **Zeaxanthin**. (a) Difference densities (ES − GS) and (b) transition densities (GS-to-ES) calculated with different methods, at the DFT B3LYP/6-31G(d) ground-state geometry. Adiabatic excited states calculated with MRSF-TDDFT have been reordered to match the other methods. The symmetry (A/B) and covalent character (+/−) of the states is evident from the transition densities.

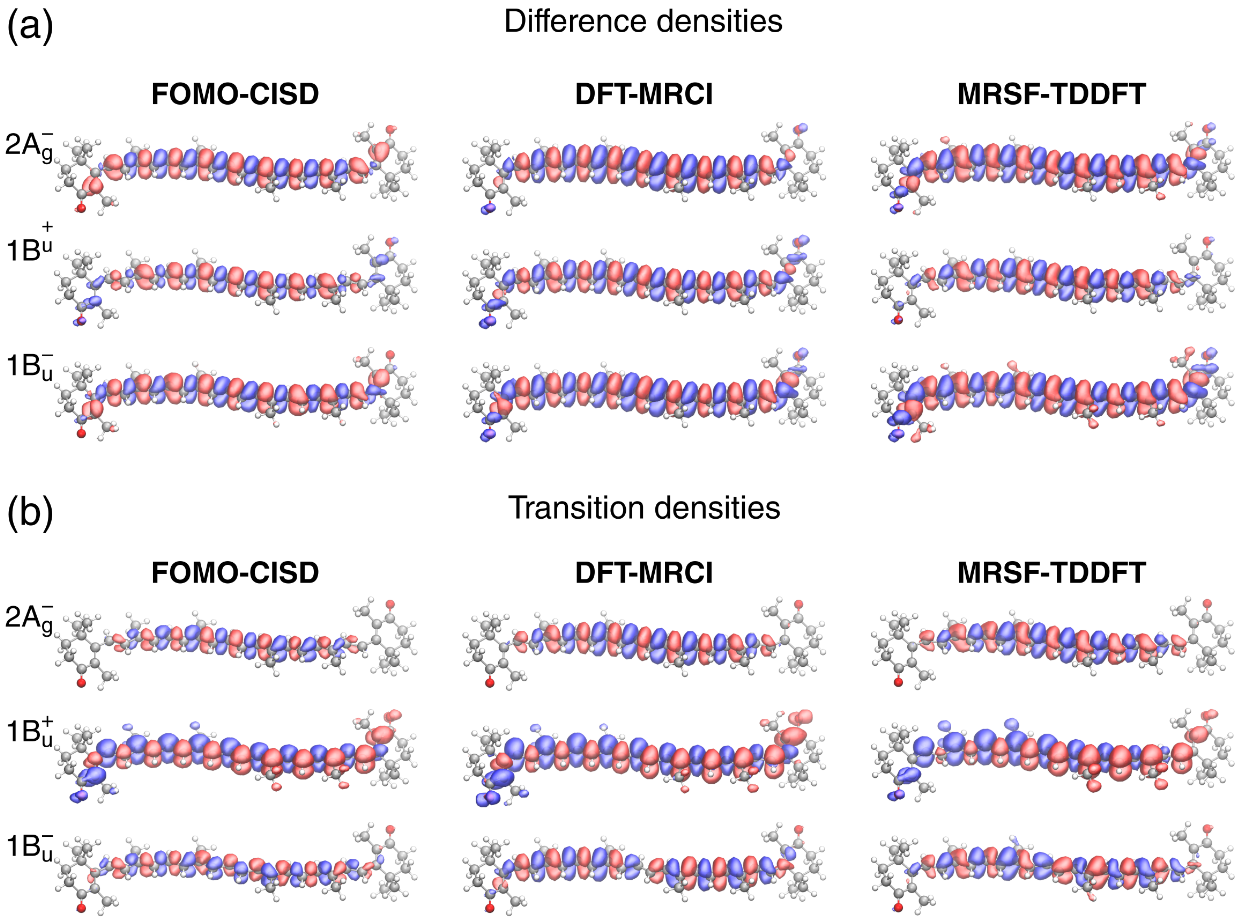

Figure S7: Excited-state characterization for **Cantaxanthin**. (a) Difference densities (ES − GS) and (b) transition densities (GS-to-ES) calculated with different methods, at the DFT B3LYP/6-31G(d) ground-state geometry. Adiabatic excited states calculated with MRSF-TDDFT have been reordered to match the other methods. The symmetry (A/B) and covalent character (+/−) of the states is evident from the transition densities.

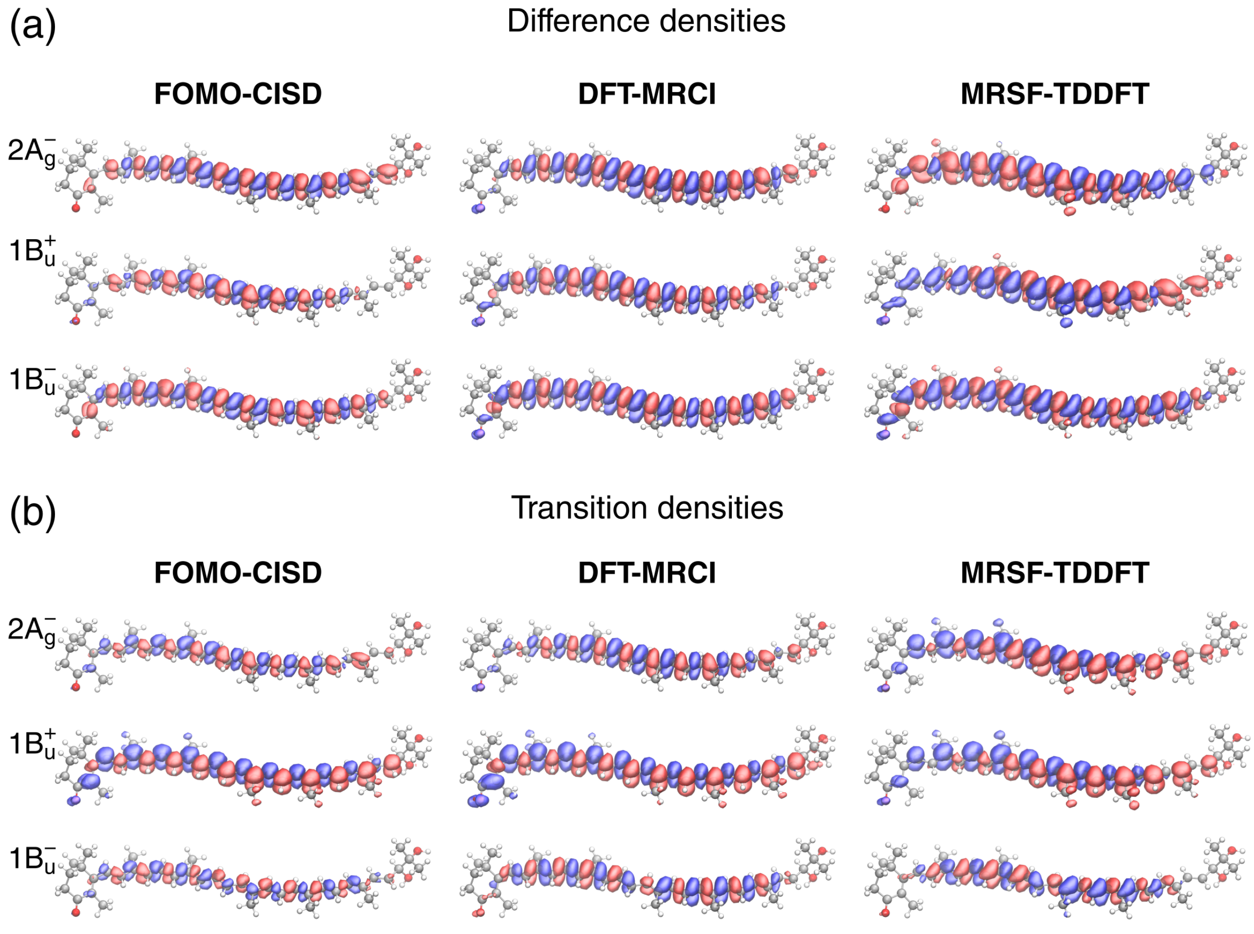

Figure S8: Excited-state characterization for **Salinixanthin**. (a) Difference densities (ES − GS) and (b) transition densities (GS-to-ES) calculated with different methods, at the DFT B3LYP/6-31G(d) ground-state geometry. Adiabatic excited states calculated with MRSF-TDDFT have been reordered to match the other methods. The symmetry (A/B) and covalent character (+/−) of the states is evident from the transition densities.

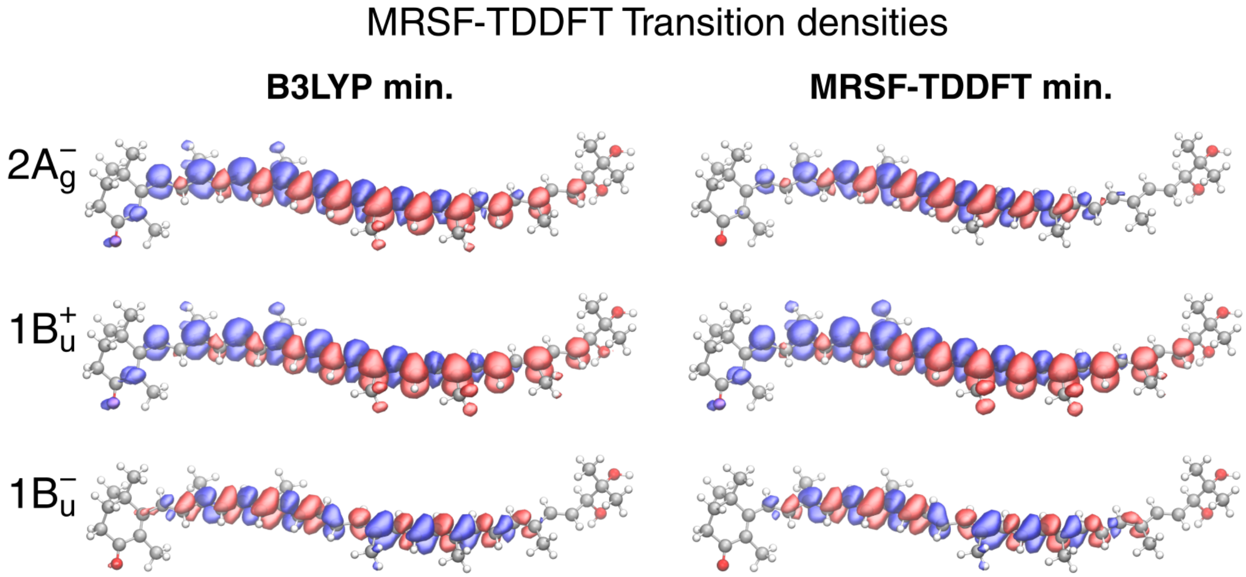

Figure S9: Comparison of transition densities (GS-to-ES) of **Salinixanthin** computed using MRSF-TDDFT/DTCAM-XIV at the optimized ground-state geometry obtained with DFT B3LYP/6-31G(d) (left) and at the MRSF-TDDFT ground-state minimum along the BLA coordinate (right).

S4 Performance of MRSF-TDDFT functionals on vertical excitation energies and BLA profiles

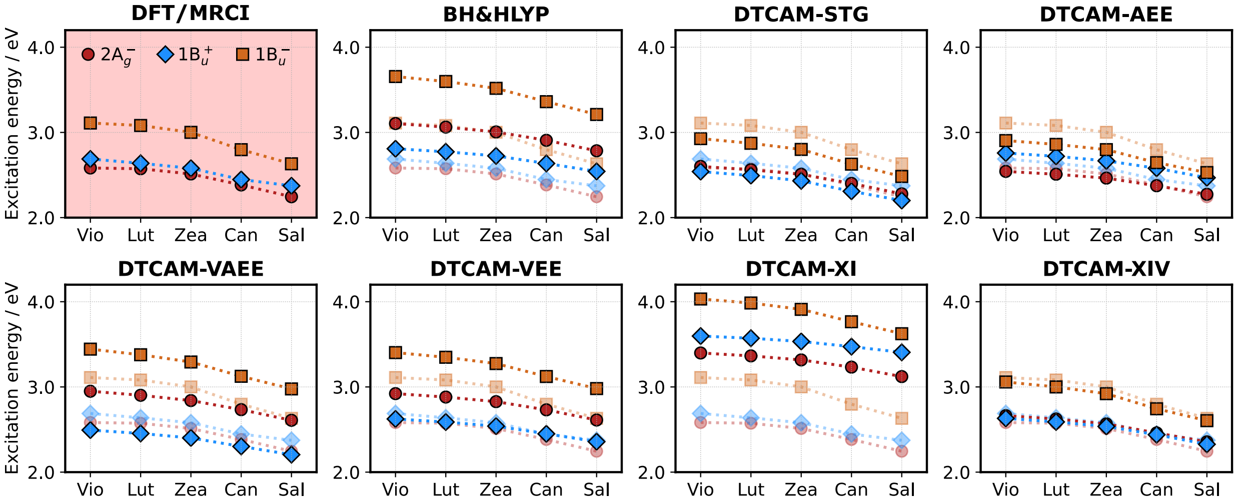

Figure S10: Vertical excitation energies computed with DFT/MRCI and MRSF-TDDFT using different DFT functionals, with the def2-SVP basis set. The DFT/MRCI/def2-SVP data is used as reference and it is reported in all plots in transparent. All ground-state geometries are optimized with B3LYP/6-31G(d).

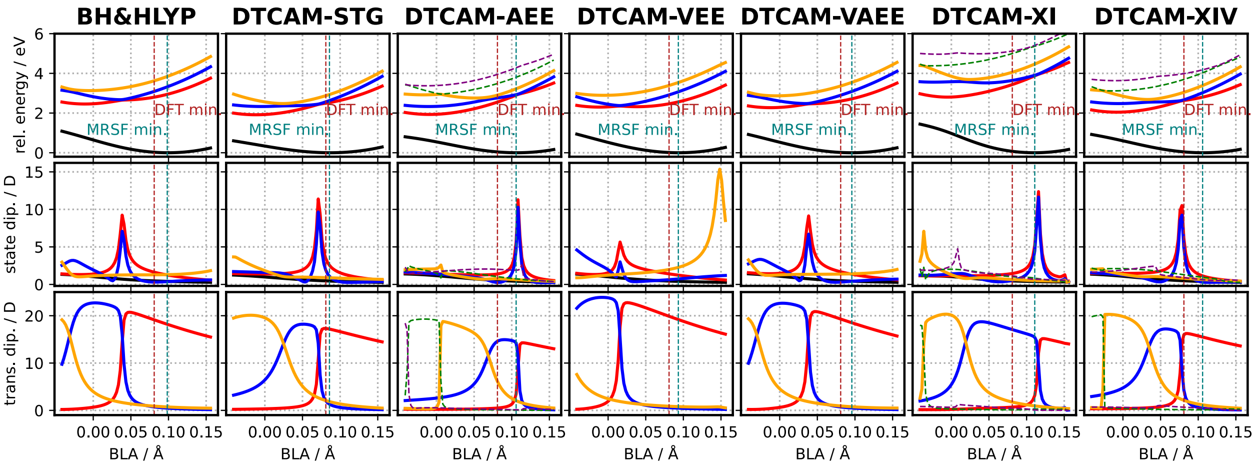

Figure S11: Relaxed scan of lutein along the BLA. Quantities computed with MRSF-TDDFT using different DFT functionals, with the def2-SVP basis set. Geometries are optimized with DFT B3LYP/6-31G(d). Top row: relative energies; middle row: state dipoles; bottom row: transition dipole moments (from the ground state).

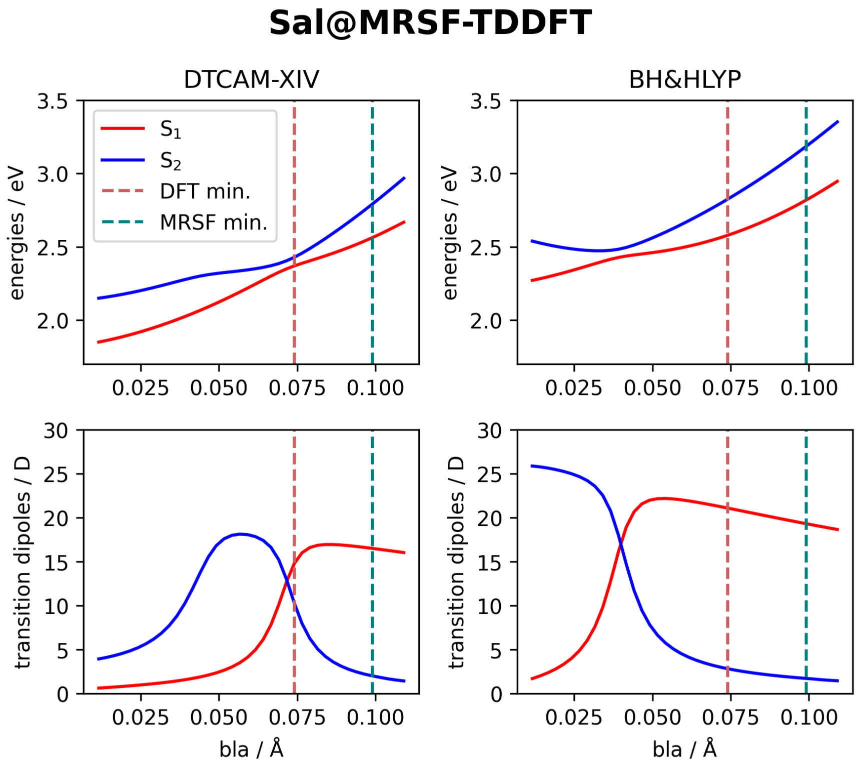

Figure S12: Zoomed-in comparison of the **Salinixanthin** S_1 and S_2 excited-state adiabatic potential energy curves (top) and transition dipole moments (bottom) computed with MRSF-TDDFT using the DTCAM-XIV (left) and BH&HLYP functionals, with the def2-SVP basis set. The red vertical dashed line indicates the DFT B3LYP/6-31G(d) optimized ground-state minimum, whereas the green dashed vertical line marks the MRSF-TDDFT ground-state minimum along the BLA coordinate (see Figure S11).

S5 Comparison of BLA potential energy curves computed with different methods

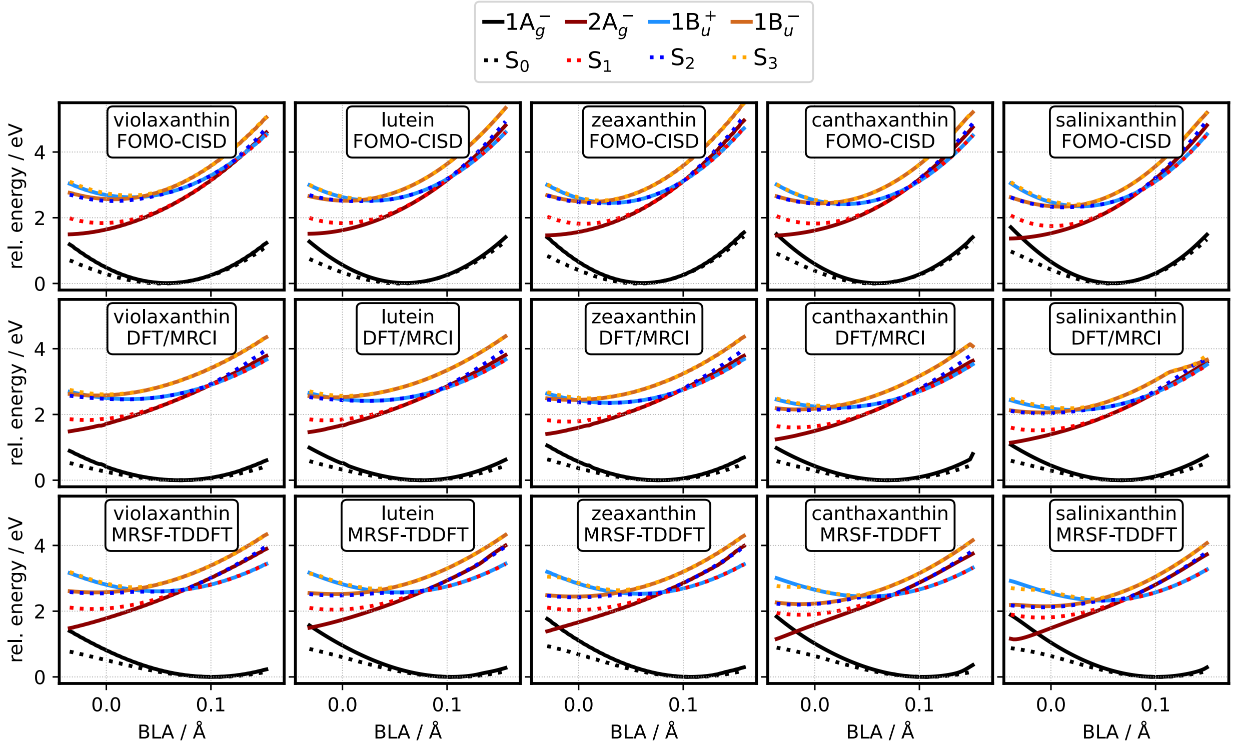

Figure S13: Relaxed PES scan along the BLA for all five xanthophylls computed with FOMO-CISD(6,9), DFT/MRCI (BH&HLYP/def2-SVP), and MRSF-TDDFT (DTCAM-XIV/def2-SVP). Geometries are optimized with DFT B3LYP/6-31G(d).

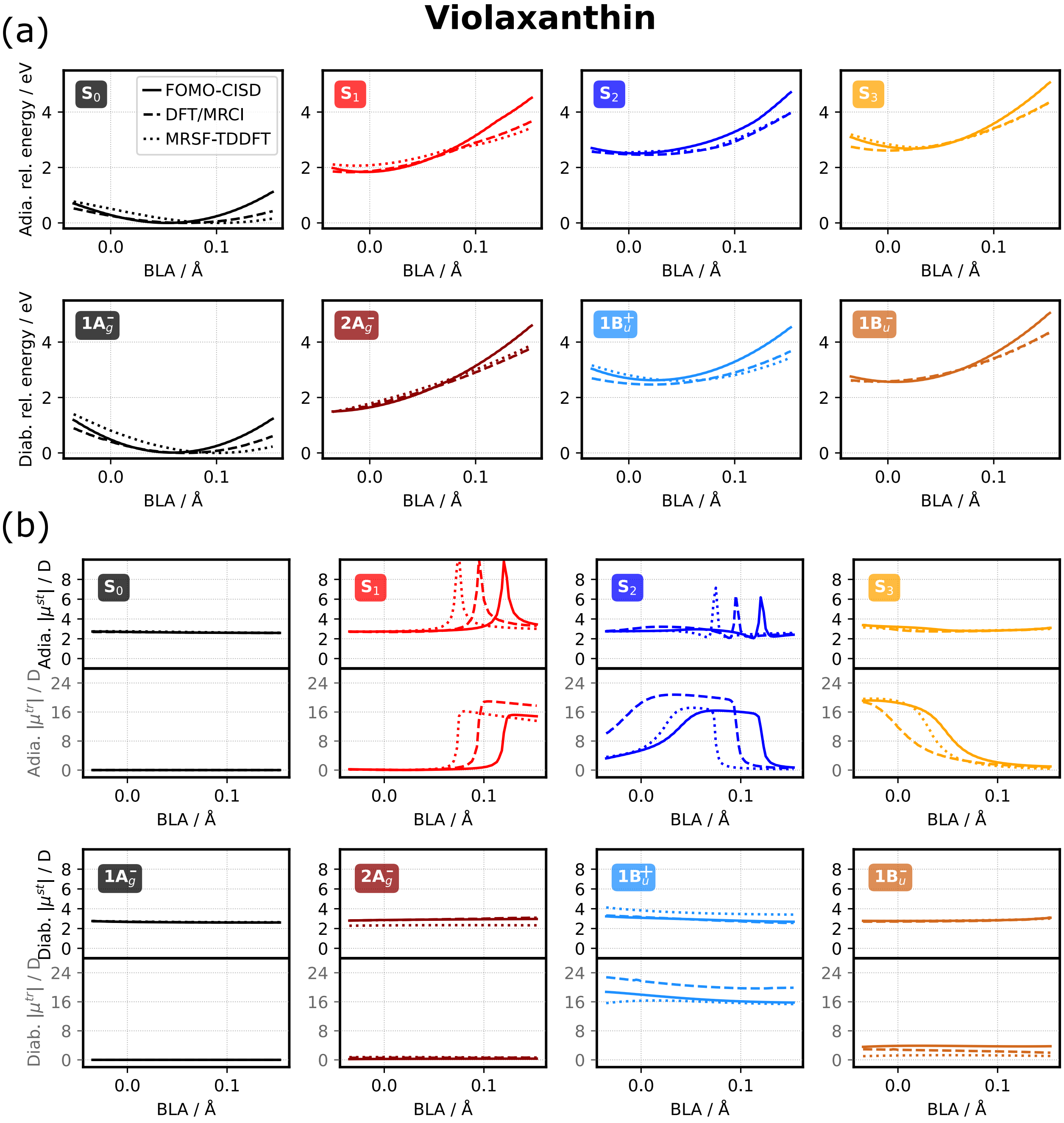

Figure S14: Violaxanthin: per-state comparison between FOMO-CISD(6,9), DFT/MRCI (BH&HLYP/def2-SVP), and MRSF-TDDFT (DTCAM-XIV/def2-SVP) along BLA relaxed scan (opt. at DFT B3LYP/6-31G(d)). (a) Energy of the states in the adiabatic and diabatic represetation. (b) State and transition dipoles in the adiabatic and diabatic representation.

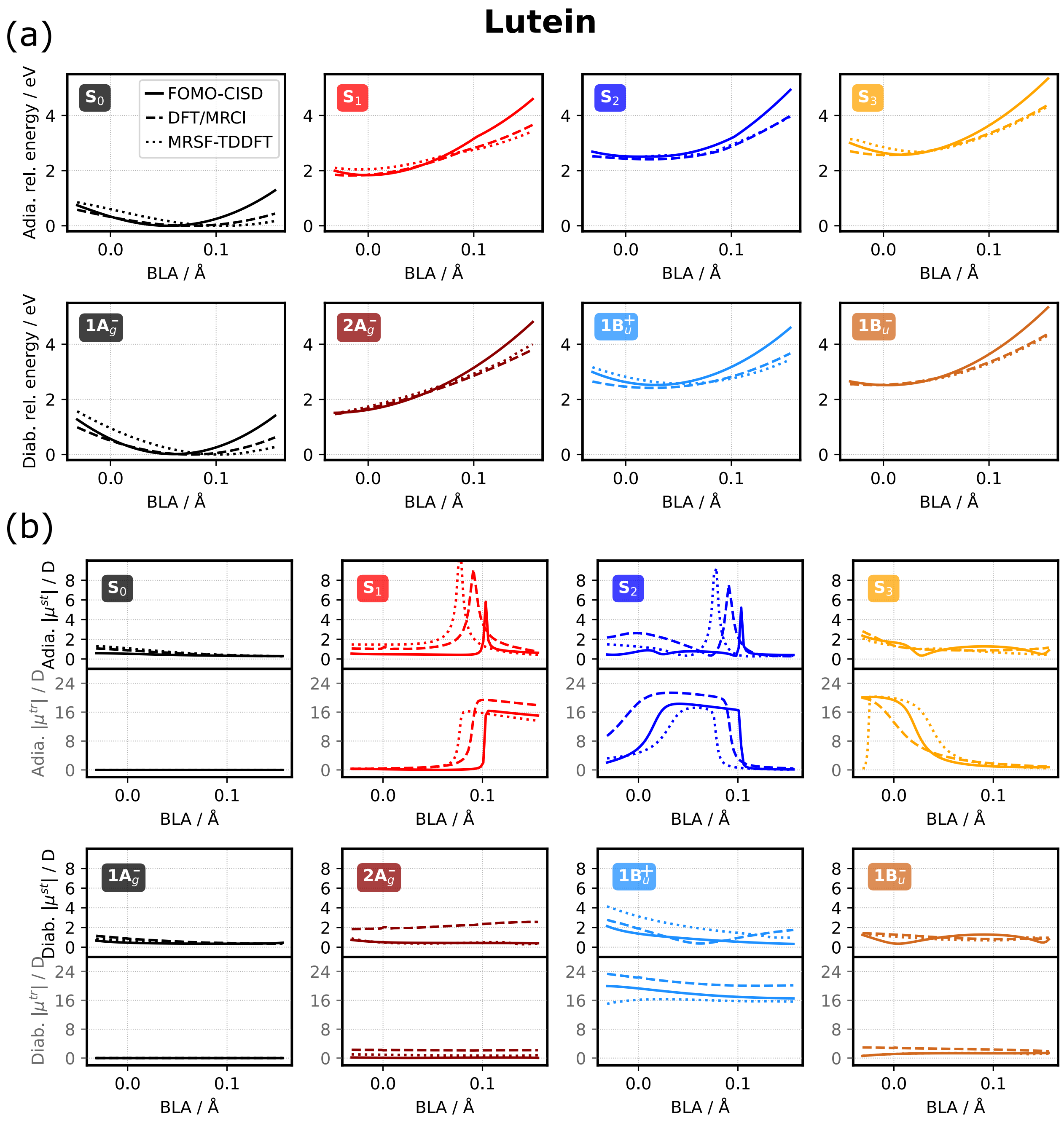

Figure S15: Lutein: per-state comparison between FOMO-CISD(6,9), DFT/MRCI (BH&HLYP/def2-SVP), and MRSF-TDDFT (DTCAM-XIV/def2-SVP) along BLA relaxed scan (opt. at DFT B3LYP/6-31G(d)). (a) Energy of the states in the adiabatic and diabatic represetation. (b) State and transition dipoles in the adiabatic and diabatic representation.

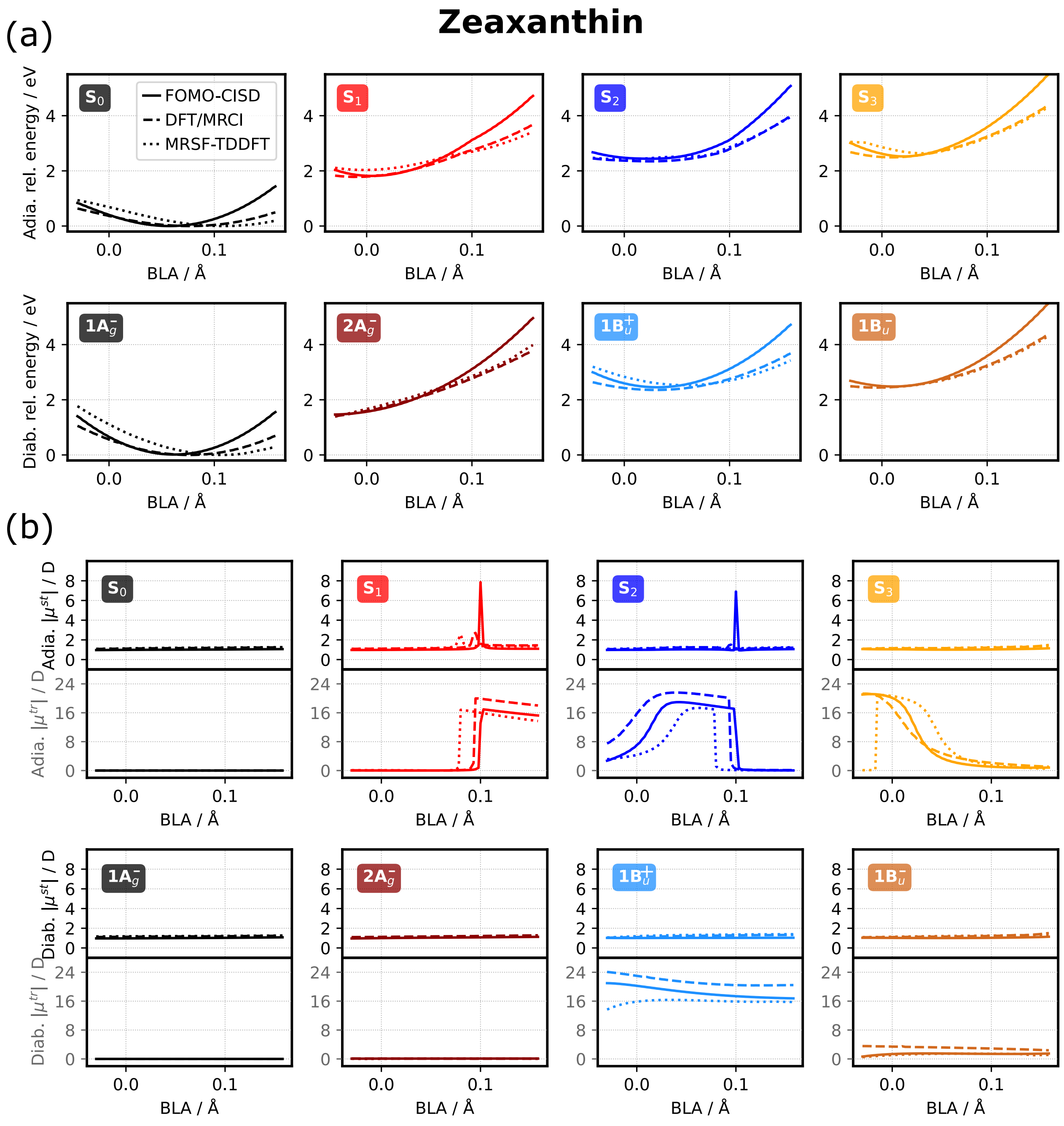

Figure S16: Zeaxanthin: per-state comparison between FOMO-CISD(6,9), DFT/MRCI (BH&HLYP/def2-SVP), and MRSF-TDDFT (DTCAM-XIV/def2-SVP) along BLA relaxed scan (opt. at DFT B3LYP/6-31G(d)). (a) Energy of the states in the adiabatic and diabatic represetation. (b) State and transition dipoles in the adiabatic and diabatic representation.

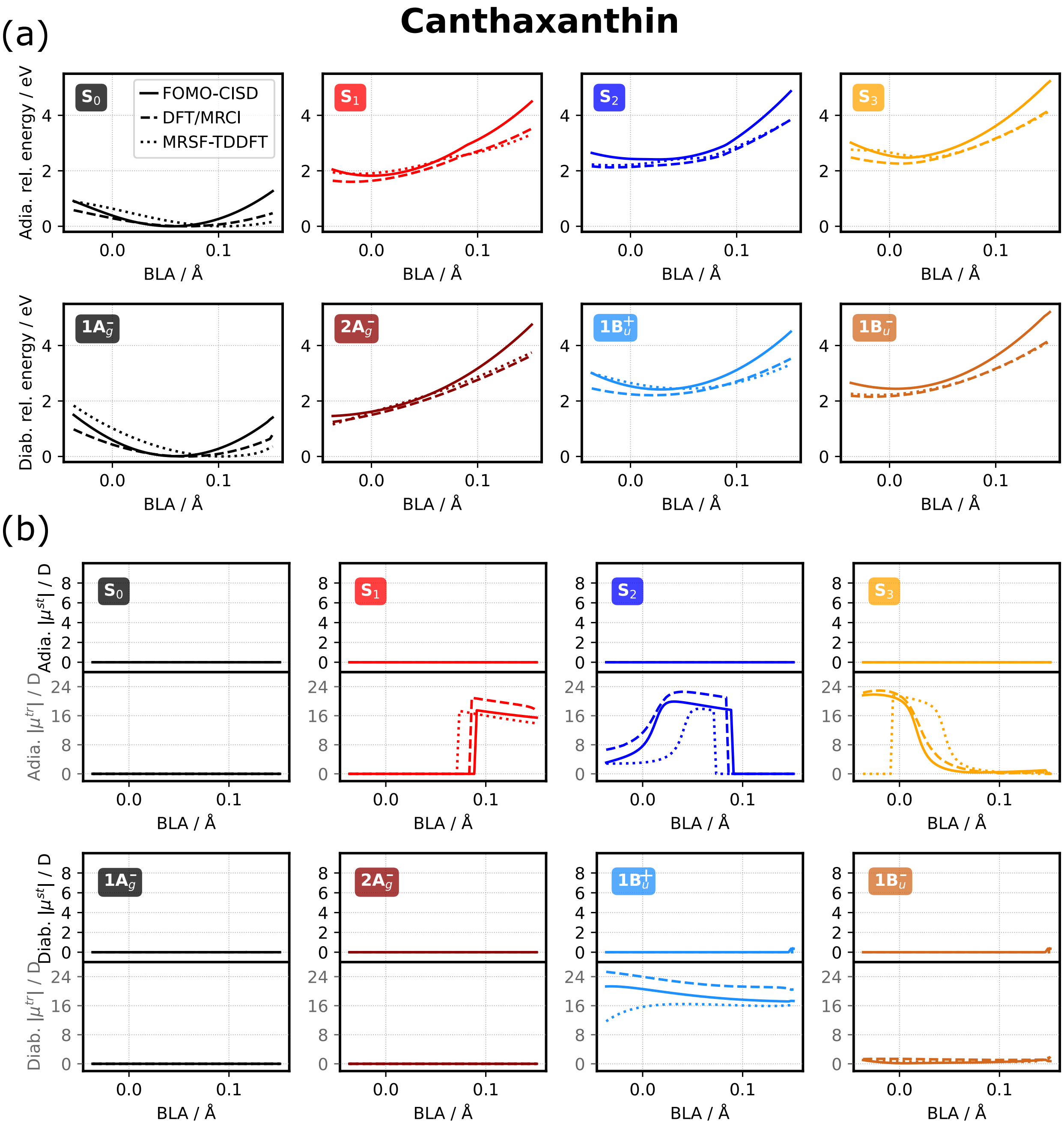

Figure S17: Canthaxanthin: per-state comparison between FOMO-CISD(6,9), DFT/MRCI (BH&HLYP/def2-SVP), and MRSF-TDDFT (DTCAM-XIV/def2-SVP) along BLA relaxed scan (opt. at DFT B3LYP/6-31G(d)). (a) Energy of the states in the adiabatic and diabatic representation. (b) State and transition dipoles in the adiabatic and diabatic representation.

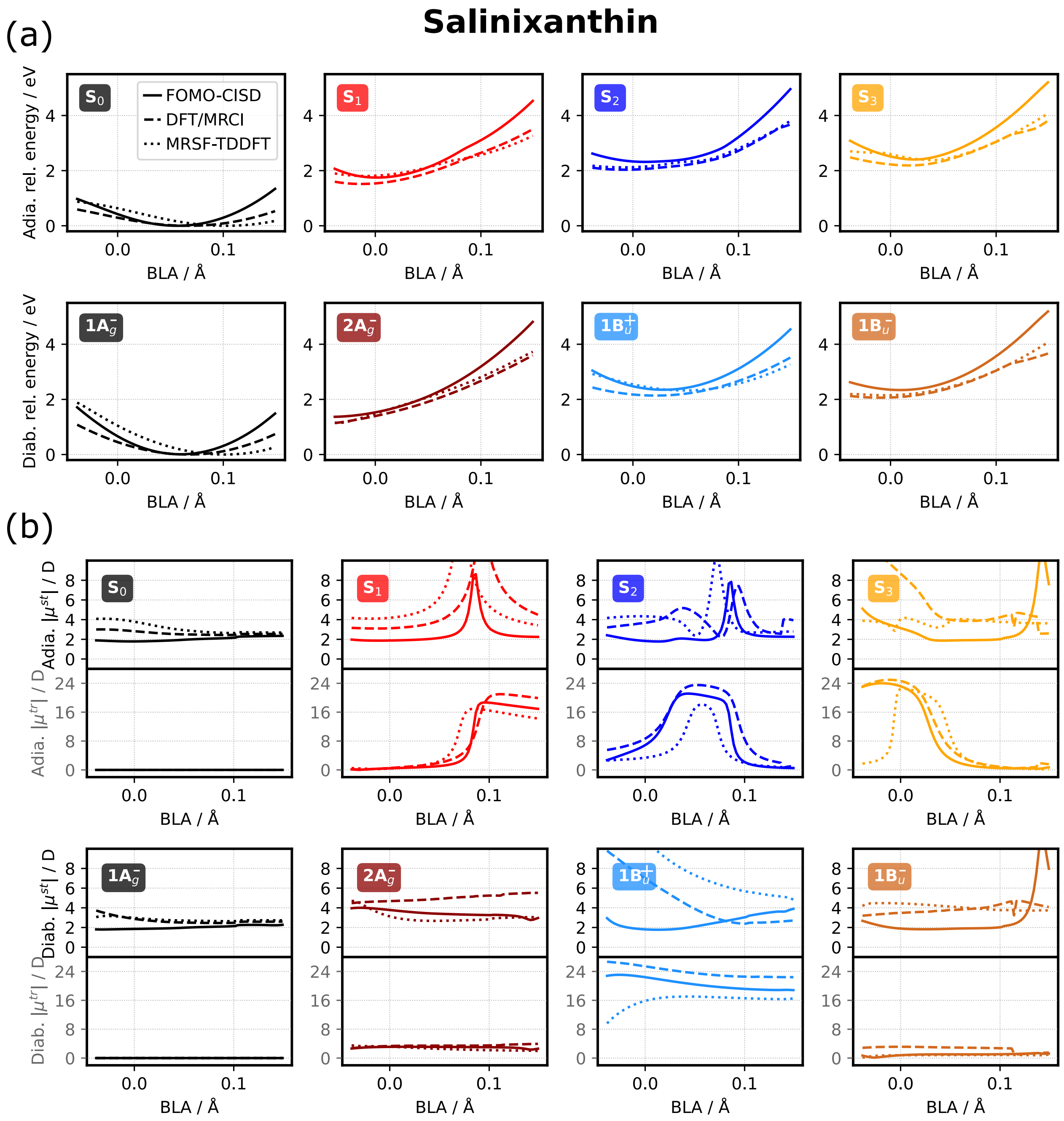

Figure S18: Salinixanthin: per-state comparison between FOMO-CISD(6,9), DFT/MRCI (BH&HLYP/def2-SVP), and MRSF-TDDFT (DTCAM-XIV/def2-SVP) along BLA relaxed scan (opt. at DFT B3LYP/6-31G(d)). (a) Energy of the states in the adiabatic and diabatic represetation. (b) State and transition dipoles in the adiabatic and diabatic representation.

S6 Raw data in vertical excitation energies

Table S1: Vertical excitation energies at the B3LYP/6-31G(d) geometries (row data of Figure 3 of the main text). In the table we report DFT/MRCI, FOMO-CISD and MRSF-TDDFT Franck Condon excitation energies and transition dipole moments for the first three excited states of the investigated carotenoids.

**Violaxanthin**						
	**FOMO-CISD**		**DFT/MRCI**		**MRSF-TDDFT**	
**state**	Exc. Ene. (eV)	Tr. Dip. (D)	Exc. Ene. (eV)	Tr. Dip. (D)	Exc. Ene. (eV)	Tr. Dip. (D)
$2A_g^-$	2.60	0.311	2.58	0.930	2.66	3.208
$1B_u^+$	2.89	16.393	2.69	19.846	2.63	16.107
$1B_u^-$	3.09	3.779	3.11	2.144	3.06	1.907
**Lutein**						
	**FOMO-CISD**		**DFT/MRCI**		**MRSF-TDDFT**	
**state**	Exc. Ene. (eV)	Tr. Dip. (D)	Exc. Ene. (eV)	Tr. Dip. (D)	Exc. Ene. (eV)	Tr. Dip. (D)
$2A_g^-$	2.63	0.113	2.57	3.044	2.62	4.318
$1B_u^+$	2.79	17.241	2.64	20.043	2.59	15.983
$1B_u^-$	3.15	1.325	3.08	2.310	3.00	2.133
**Zeaxanthin**						
	**FOMO-CISD**		**DFT/MRCI**		**MRSF-TDDFT**	
**state**	Exc. Ene. (eV)	Tr. Dip. (D)	Exc. Ene. (eV)	Tr. Dip. (D)	Exc. Ene. (eV)	Tr. Dip. (D)
$2A_g^-$	2.61	0.094	2.51	0.170	2.57	0.416
$1B_u^+$	2.74	17.693	2.58	20.496	2.54	16.747
$1B_u^-$	3.11	1.430	3.00	2.696	2.92	2.397
**Canthaxanthin**						
	**FOMO-CISD**		**DFT/MRCI**		**MRSF-TDDFT**	
**state**	Exc. Ene. (eV)	Tr. Dip. (D)	Exc. Ene. (eV)	Tr. Dip. (D)	Exc. Ene. (eV)	Tr. Dip. (D)
$2A_g^-$	2.57	0.000	2.38	0.000	2.46	0.000
$1B_u^+$	2.67	18.115	2.45	21.368	2.44	17.198
$1B_u^-$	3.04	0.377	2.80	0.801	2.74	1.344
**Salinixanthin**						
	**FOMO-CISD**		**DFT/MRCI**		**MRSF-TDDFT**	
**state**	Exc. Ene. (eV)	Tr. Dip. (D)	Exc. Ene. (eV)	Tr. Dip. (D)	Exc. Ene. (eV)	Tr. Dip. (D)
$2A_g^-$	2.52	3.061	2.25	4.773	2.37	10.283
$1B_u^+$	2.61	19.694	2.37	22.538	2.31	14.789
$1B_u^-$	2.91	1.026	2.63	2.181	2.60	2.062

S7 Experimental data on the bright state ($1B_u^+$)

Table S2: Collection of experimental observations of the vertical excitation energy (in eV) of the bright state ($1B_u^+$) via spectroscopic measurements in different solvents.

	**Vio**	**Lut**	**Zea**	**Can**	**Sal**
N-Hexane	-	-	2.76 [S1]	-	2.42 [S2]
Toluene	-	2.71 [S1]	2.68 [S1]	2.64 [S1]	-
Chloroform	-	2.72 [S1]	2.68 [S1]	2.64 [S1]	-
DMSO	-	2.68 [S1]	-	-	-
Acetone	-	2.77 [S1]	2.74 [S1]	2.70 [S1]	-
Acetonitrile	-	2.77 [S1]	2.70 [S1]	2.70 [S1]	-
Ethanol	-	2.77 [S1]	2.74 [S1]	-	-
Methanol	-	2.79 [S1] 2.63 [S3]	2.76 [S1] 2.60 [S3]	-	2.46 [S2]
EPA	2.56 [S4]	2.52 [S4]	2.48 [S4]	-	-

S8 Effect of the optimization on MRSF-TDDFT excitation energies

Table S3: Excited states of all xanthophylls at the MRSF-TDDFT level with two different functionals (BH&HLYP and DTCAM-XIV) at the MRSF-TDDFT ground-state geometry optimized with the same functional. Excitation energies (eV), transition dipole moments (D), and BLA values (Å).

func.	**BH&HLYP**					**DTCAM-XIV**				
	S_1 ene.	S_2 ene.	S_1 μ_{tr}	S_2 μ_{tr}	BLA	S_1 ene.	S_2 ene.	S_1 μ_{tr}	S_2 μ_{tr}	BLA
Can	2.902	3.289	18.469	0.000	0.097	2.815	2.974	15.473	0.000	0.104
Lut	2.986	3.382	17.990	0.414	0.099	2.883	3.047	15.232	0.202	0.106
Sal	2.818	3.169	19.358	1.273	0.096	2.752	2.908	15.854	0.534	0.103
Vio	3.005	3.406	17.875	0.373	0.096	2.894	3.058	15.195	0.581	0.103
Zea	2.953	3.341	18.203	0.081	0.101	2.854	3.016	15.334	0.031	0.108

S9 Comparison with *ab initio* multireference calculations

In this section we analyze the adiabatic energies of the first two excited states, $2A_g^-$ and $1B_u^+$. The adiabatic energies are defined as the difference of the energy of the excited state in its minimum and the energy of ground state in its minimum (Figure S19). The calculations that we report are done on the geometries of Ref.,[S5] as we can compare our results with their DSRG-MRPT2 calculations. The adiabatic energies are reported in Table S4. To be thorough, we also report in Table S5 the vertical excitation energies of all states (S_1, S_2 and S_3) at the three different minima ($1A_g^-$, $2A_g^-$ and $1B_u^+$). We note that these calculations do not have a direct comparison with the data extracted from Ref.,[S5] therefore we focus on analyzing the adiabatic energies. We can see from Table S4 that the bright state adiabatic energy is well reproduced by all methods for all carotenoids. When it comes to the dark state, on the other hand, there are far more discrepancies between the methods. In this case, the MRSF-TDDFT values are the ones that closer resemble the DSRG-MRPT2 reference. DFT/MRCI only slightly underestimates the $2A_g^-$ value, while the FOMO-CISD strongly underestimates it, in particular for lutein and violaxanthin. Zeaxanthin, indeed, shows the best agreement between the methods.

In order to understand this behavior, we have to observe the value of the BLA in the two minima. The BLA of the $1B_u^+$ minimum is closer to the one of the ground state in all cases (which is 1.00 for Vio, 1.00 for Lut and 1.05 for Zea). The BLA of the $2A_g^-$ minimum is instead strongly shifted towards small, even negative BLA values. This means that the accuracy of the methods in reproducing also the ground state energy at lower BLA is affecting the adiabatic energy results. In FOMO-CISD, in particular, the minimum BLA of the ground state is far from the one predicted with this geometry optimization method, which affects the relative position of the minima of the two states (Figure S13).

Something that has to be noted here is that for the evaluation of a quantity such as the

adiabatic energies, the optimization method employed is a crucial aspect, as it also affects the location of the ground state minimum. We have no guarantee that the methodologies adopted for the ground and excited state optimizations is ideal for all the investigated methods. The best performance of MRSF-TDDFT, from this perspective, is probably due to a better compatibility between the optimization methodology and the excited state method. This is reinforced by the fact that the MRSF-TDDFT minimum BLA for the ground state is basically the same as the CAM-B3LYP one. In this context, the adiabatic energy should be evaluated for each method starting from a geometry optimization performed with the same method.

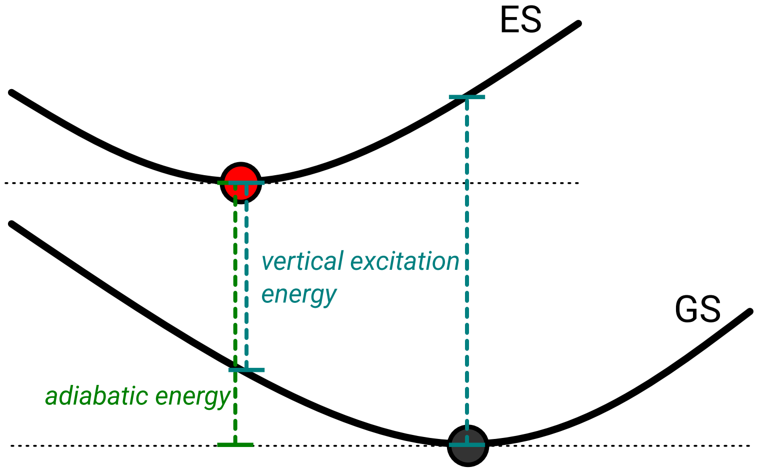

Figure S19: Scheme representing the definition of vertical excitation energy and adiabatic energy.

Table S4: Adiabatic excitation energies for the $2A_g^-$ and $1B_u^+$ states computed with DFT/MRCI, FOMO-CISD and MRSF-TDDFT and compared with DSRG-MRPT2 results from Ref.[S5] The geometries are the ones from Ref.[S5] and were optimized as follows: $1A_g^-$ with DFT/CAM-B3LYP/cc-pVDZ, $2A_g^-$ with SF-TD-DFT/BH&HLYP/cc-pVDZ and $1B_u^+$ with TD-DFT/CAM-B3LYP/cc-pVDZ. The value of BLA is also reported.

**Violaxanthin**					
min.	**DSRG-MRPT2**	**FOMO-CISD**	**DFT/MRCI**	**MRSF-TDDFT**	BLA
$2A_g^-$	2.05	1.04	1.70	1.92	-0.004
$1B_u^+$	2.58	2.60	2.43	2.57	0.038
**Lutein**					
min.	**DSRG-MRPT2**	**FOMO-CISD**	**DFT/MRCI**	**MRSF-TDDFT**	BLA
$2A_g^-$	1.96	1.00	1.70	1.91	0.005
$1B_u^+$	2.50	2.43	2.36	2.53	0.044
**Zeaxanthin**					
min.	**DSRG-MRPT2**	**FOMO-CISD**	**DFT/MRCI**	**MRSF-TDDFT**	BLA
$2A_g^-$	1.89	1.71	1.70	2.00	0.008
$1B_u^+$	2.42	2.37	2.30	2.61	0.049

Table S5: Vertical excitation energies calculated on the geometries of Ref.,[S5] optimized as follows: $1A_g^-$ with DFT/CAM-B3LYP/cc-pVDZ, $2A_g^-$ with SF-TD-DFT/BH&HLYP/cc-pVDZ and $1B_u^+$ with TD-DFT/CAM-B3LYP/cc-pVDZ. In the table we report the excitation energy of the first three excited states at the minimum geometry of all three states. The energies are computed with DFT/MRCI, FOMO-CISD and MRSF-TDDFT. The value of BLA is also reported. The state with the larges transition dipole moment is highlighted in bold. The excitation energies available from Ref.[S5] are compared with our results in Table 1a of the main text.

**Violaxanthin**										
	**FOMO-CISD**			**DFT/MRCI**			**MRSF-TDDFT**			
min.	S_1	S_2	S_3	S_1	S_2	S_3	S_1	S_2	S_3	BLA
$1A_g^-$	2.94	**3.06**	3.36	**2.86**	2.91	3.39	**2.82**	3.05	3.43	0.100
$2A_g^-$	1.44	2.21	**2.43**	1.53	**2.21**	2.36	1.50	2.04	**2.38**	−0.004
$1B_u^+$	1.95	2.53	**2.62**	2.00	**2.40**	2.65	2.00	**2.37**	3.51	0.038
**Lutein**										
	**FOMO-CISD**			**DFT/MRCI**			**MRSF-TDDFT**			
	S_1	S_2	S_3	S_1	S_2	S_3	S_1	S_2	S_3	BLA
$1A_g^-$	**2.97**	3.04	3.90	**2.82**	2.91	3.37	**2.80**	3.03	3.40	0.100
$2A_g^-$	1.00	1.83	**2.32**	1.50	**2.14**	2.30	1.43	1.94	**2.31**	0.005
$1B_u^+$	1.93	**2.48**	3.00	1.98	**2.33**	2.61	1.94	**2.30**	2.44	0.044
**Zeaxanthin**										
	**FOMO-CISD**			**DFT/MRCI**			**MRSF-TDDFT**			
	S_1	S_2	S_3	S_1	S_2	S_3	S_1	S_2	S_3	BLA
$1A_g^-$	**2.93**	2.98	3.44	**2.78**	2.87	3.32	**2.76**	3.00	3.35	0.105
$2A_g^-$	1.40	2.10	**2.23**	1.42	**2.02**	2.15	1.34	1.76	**2.19**	0.008
$1B_u^+$	1.95	**2.42**	2.56	1.93	**2.28**	2.53	1.90	2.24	**2.38**	0.049

S10 Diabatization analysis

S10.1 Choosing the diabatization reference

The diabatization of the states along the scan of the bond length alternation (BLA) can be performed by choosing an arbitrary reference, as long as the reference states are well separated, weakly mixed, and exhibit the expected ordering (with the $1A_g^-$ state lying below the $1B_u^+$ state). In the results reported in the main text, we always used as reference the geometry corresponding to the minimum of the BLA scan of each method.

In order to assess the effect of the reference geometry on the diabatization, we report canthaxanthin and lutein at the FOMO-CISD level as test cases. We optimized the geometry of the two selected carotenoids at the FOMO-CISD level, as well as at the DFT CAM-B3LYP level and DFT B3LYP level. The latter method was also used to perform the relaxed scan over the BLA, and therefore the opmized geometry coincides with the B3LYP ground state minimum of the scan. For each of these geometries, we computed the electronic states at the FOMO-CISD level. Then we compared the diabatic states obtained using these different references, namely FOMO-CISD, DFT/B3LYP and DFT/CAM-B3LYP, to the minimum BLA reference used in the main text. In Figure S20 and Figure S21 we show the energies, dipoles and transition dipoles of the diabatic states obtained with the different references, whose BLA value is highlighted with the violet line. Notably, all transformations yield qualitatively similar results. Small differences are observed due to changes in the reference properties, which produce slightly different adiabatic-to-diabatic transformation matrices. As can be seen from the dipoles, in all cases the diabatization is successful in removing the abrupt changes in electronic character. The magnitude of state and transition dipoles is similar for all references. The largest discrepancy is found for the properties of the $1B_u^-$ state, which likely has different degrees of mixing with the $1B_u^+$ state at the different reference geometries.

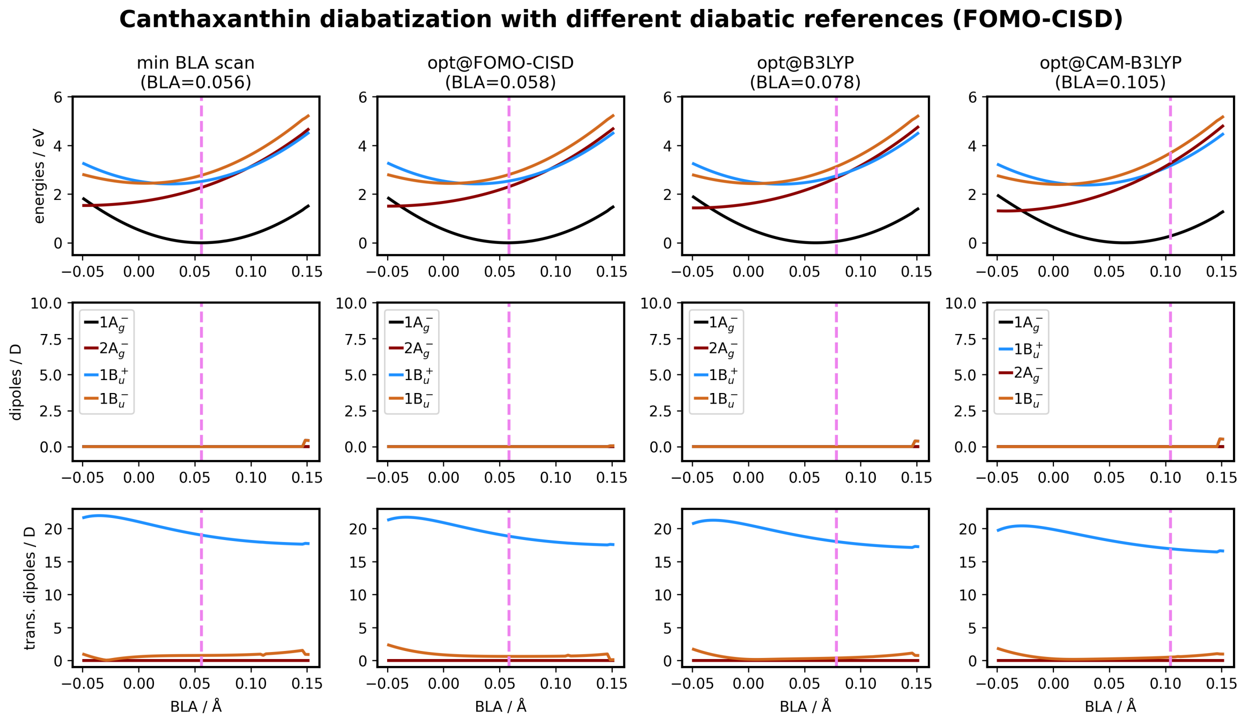

Figure S20: Diabatic states of **Canthaxanthin** obtained with different references. The reference BLA is highlighted with the violet line.

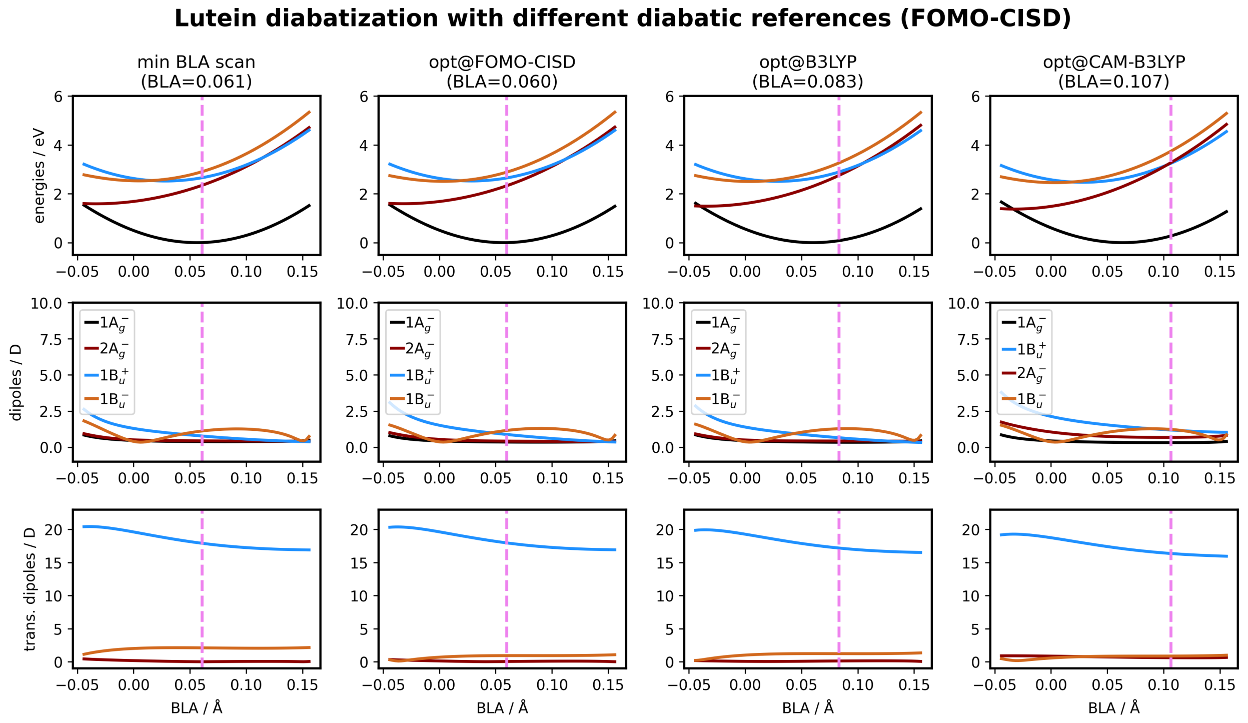

Figure S21: Diabatic states of **Lutein** obtained with different references. The reference BLA is highlighted with the violet line.

S10.2 Choosing the optimal number of states

We analyzed the results of the diabatization of cantanxanthin (Can) in OCP (both for the vacuum states and environment states computed at the FOMO-CISD level) in order to establish the optimal diabatization strategy. The label VAC-Can@OCP indicates the vacuum calculations of Can at the OCP geometry, while OCP-Can@OCP indicates the environment calculations of Can at the OCP geometry (same as Figure 7 in the main text). In Figure S22a we analyze the vacuum case. In this test, we included more electronic states in the diabatization (up to S_7) and observed the transformation matrices along the BLA.

At BLA = 0.07 Å the states are very similar to the reference and the transformation matrix is close to the identity, except for very high energy states. In all the other cases, instead, we can identify two blocks of states mixing with each other. One is the S_0-S_3, where off-diagonal elements represent the exchange of those states along the BLA and the mixing of S_1 with the ground state. The other block includes the states S_4-S_7, that are all strongly mixed with each other. The off-diagonal blocks are almost always close to zero. We can observe some relevant mixing between S_5 and S_0 at lower BLA values, but since those states are very far in energy, this mixing is probably an artifact of the diabatization due to similarities in the states properties. States at the interface between the two blocks, S_3 and S_4, are instead very weakly coupled. For this reason, we decided to always exclude the states above S_3 in the vacuum ATD transformation.

In Figure S22b we show the same thing but this time for the environment states that are transformed into diabatic using as reference the optimized geometry of Can in vacuum. This corresponds to the 1-step environment ATD transformation. Comparing the transformation matrices with the ones of the vacuum case, we see that the mixing between the two blocks increases. It is particularly evident for the BLA = 0.11 Å case, where the term that couples S_3 and S_4 is significantly larger, as well as in the BLA = 0.07 Å case, where we see some differences also for the S_2–S_4 coupling. This means that in the environment case, it is not a good approximation to truncate at the fourth excited state. Since this additional mixing is

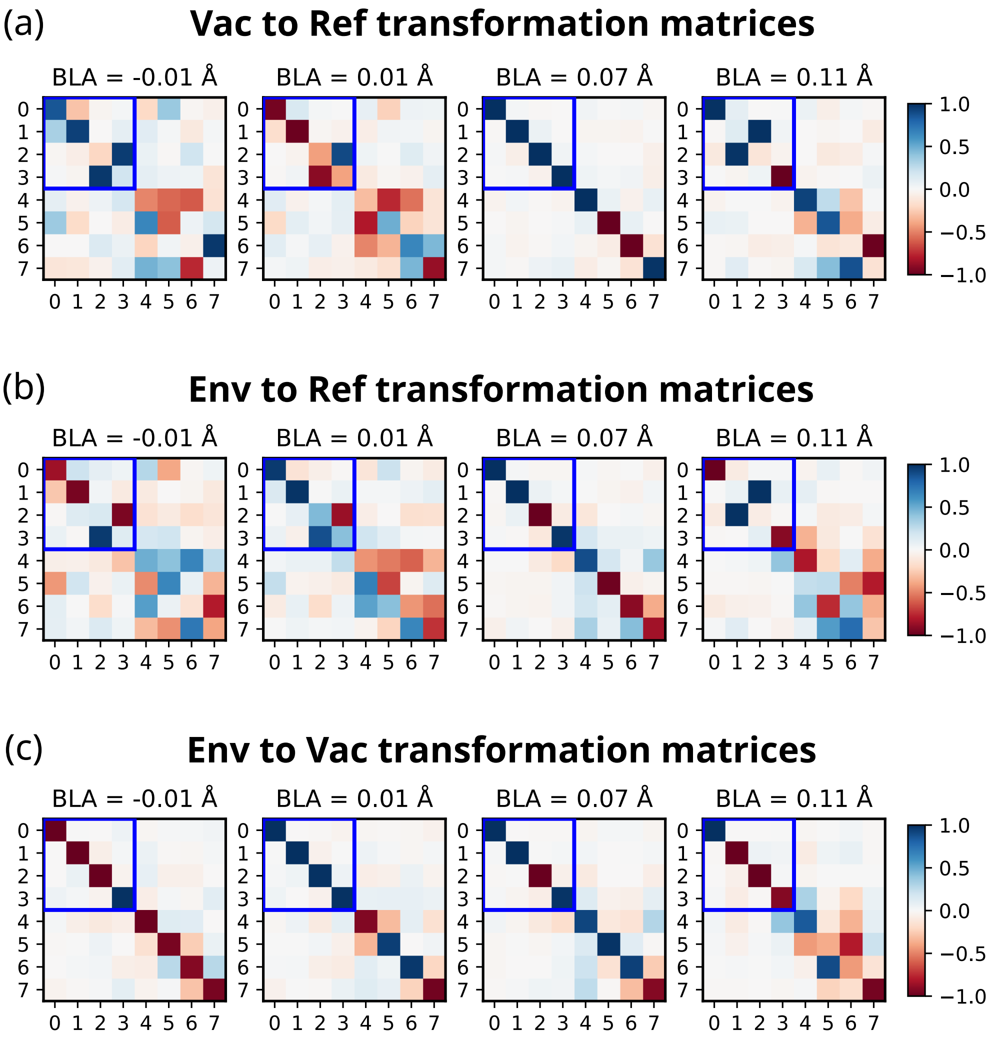

Figure S22: Transformation matrices found with the MPB method. The transformation is done including 8 electronic states. The blue square highlights the most relevant states (S_0-S_3). (a) Transformation matrices of VAC-Can@OCP states (at different BLA values) using the gas-phase optimized geometry of Can as reference. (b) Transformation matrices of OCP-Can@OCP states optimized geometry (at different BLA values) using the gas-phase optimized geometry of Can as reference. (c) Transformation matrices of environment states of Can at the OCP optimized geometry (at different BLA values) using the corresponding vacuum states of Can as reference.

only introduced by the environment, it can be removed with the rotation of the environment states to resemble the corresponding vacuum states (Figure S22c). In other words, we isolate the effect of the environment from the effect of the geometry and only observe the mixing induced by the environment. We can therefore perform a first transformation in which the states are transformed in a vacuum-like representation including the larger number of states,

and then perform the adiabatic to diabatic transformation with 4 states, as in the vacuum case.

In order to evaluate the performance of the diabatization and to determine which is the optimal number of states that need to be included, we compute the cosine similarity between the reference properties ($\mathbf{R}_p$) and the transformed properties ($\mathbf{T}_p$), summing over all the properties p used for the diabatization:

$$\mathrm{s}_{\cos} = \sum_{p=1}^{n_p} \frac{\mathbf{R}_p \cdot \mathbf{T}_p}{||\mathbf{R}_p||^2||\mathbf{T}_p||^2} \tag{S1}$$

We noticed that even small deviations from 1 in the cosine similarity could be significant for evaluating the performance of the diabatization. For this reason, we also assess the resulting diabatic potential energy curvesn(PECs) in order to analyze whether discontinuities are introduced by the diabatization.

In Figure S23 we see that in the vacuum case including states over S_3 does not improve the diabatization. The inclusion of higher energy states lead to a strong decrease in the cosine similarity, and the PECs of the lower states are worsened by addition of other states. This confirms that the 4-state diabatization is the most robust for carotenoids in vacuo. In Figure S24 we see the same thing for the environment case. However, also in the 4-states diabatization there is a decrease of the cosine similarity in the higher BLA region with respect to the vacuum case. This is solved including more states, but this would instead worsen the diabatization at small BLA values. We can solve this by performing the diabatization in two steps. In the first step, we increase the number of states because we always have a close reference (the corresponding vacuum states for each geometry). In the second step, we limit to the 4-states model. In this way we see an improvement in the cosine similarity without introducing discontinuities in the PECs (Figure S26), as we would using many states also in the second step (Figure S25).

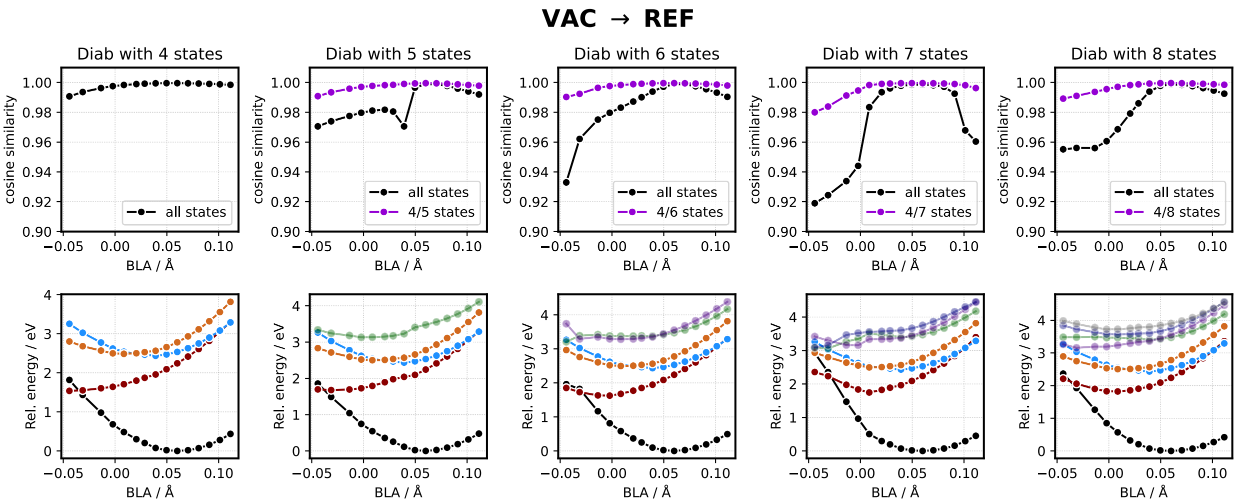

Figure S23: Top row: Cosine similarity of the MPB diabatization of VAC-Can@OCP with respect to the gas-phase optimized geometry of Can. The black plot is the cosine similarity computed including all the states used in the diabatization; the purple plot is the cosine similarity computed for the first 4 states. Bottom row: diabatic energies.

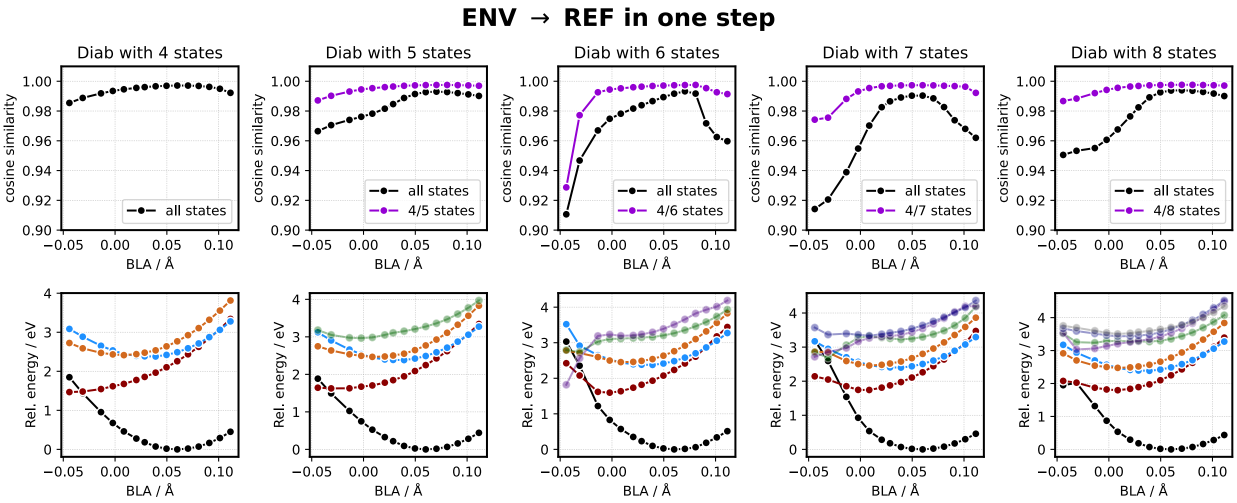

Figure S24: Top row: Cosine similarity of the MPB diabatization of OCP-Can@OCP in one step with respect to the gas-phase optimized geometry of Can. The black plot is the cosine similarity computed including all the states used in the diabatization; the purple plot is the cosine similarity computed for the first 4 states. Bottom row: diabatic energies.

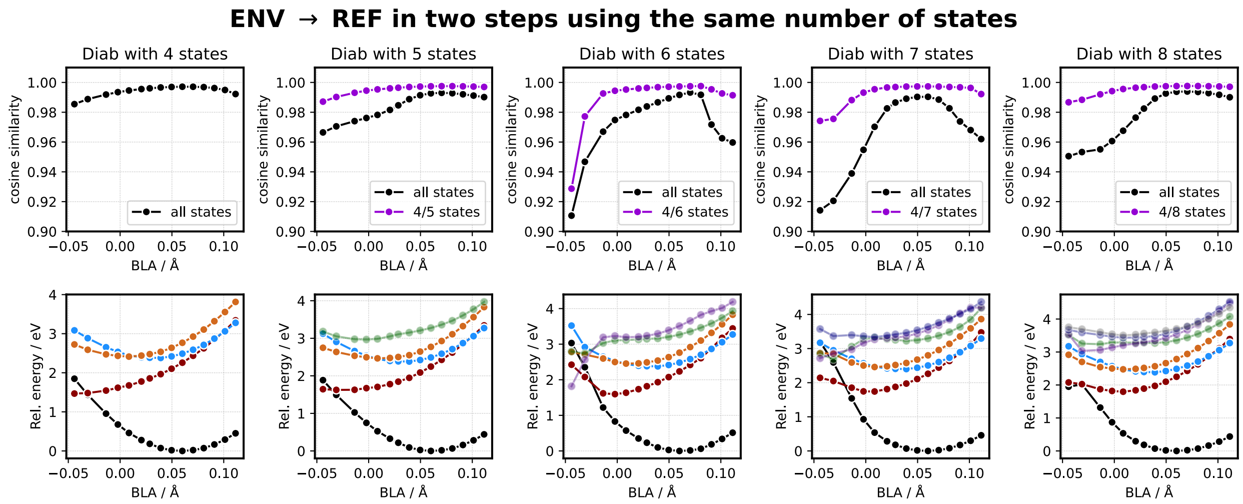

Figure S25: Top row: Cosine similarity of the MPB diabatization of OCP-Can@OCP in two steps with respect to the correspondent VAC-Can@OCP states in the first step and the states of the gas-phase optimized geometry of Can in the second step. The number of states in the two steps is the same. The black plot is the cosine similarity computed including all the states used in the diabatization; the purple plot is the cosine similarity computed for the first 4 states. Bottom row: diabatic energies.

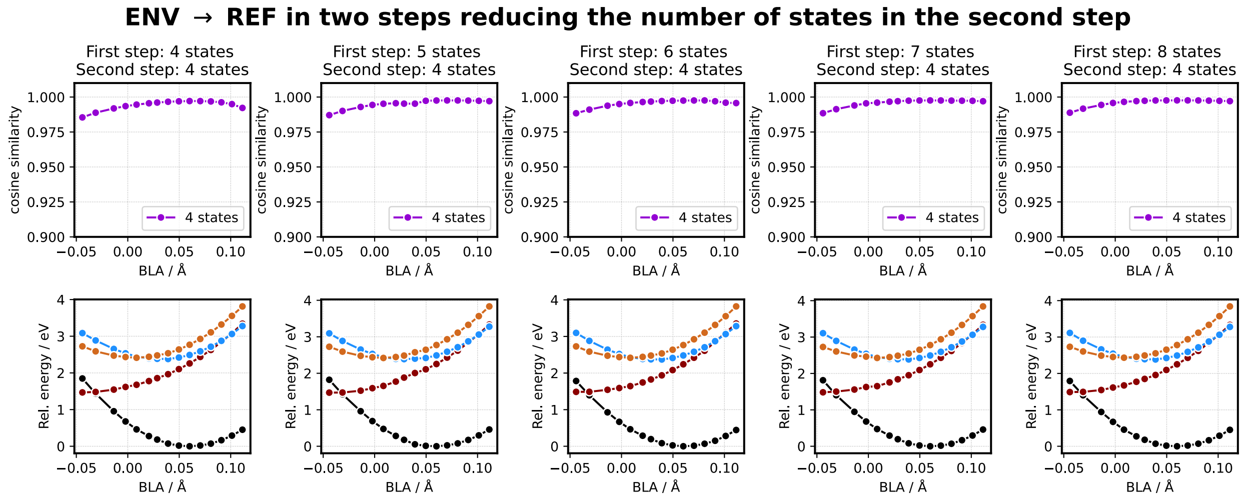

Figure S26: Top row: Cosine similarity of the MPB diabatization of OCP-Can@OCP in two steps with respect to the correspondent VAC-Can@OCP states in the first step and the states of the gas-phase optimized geometry of Can in the second step. The number of states changes in the first step but is always 4 in the second step. The cosine similarity is always computed for 4 states. Bottom row: diabatic energies.

References

(S1) Kopczynski, M.; Lenzer, T.; Oum, K.; Seehusen, J.; Seidel, M. T.; Ushakov, V. G. Ultrafast transient lens spectroscopy of various C40 carotenoids: lycopene, β-carotene, (3R, 3'R)-zeaxanthin, (3R, 3'R, 6'R)-lutein, echinenone, canthaxanthin, and astaxanthin. *Phys. Chem. Chem. Phys.* **2005**, *7*, 2793.

(S2) Polívka, T.; Balashov, S. P.; Chábera, P.; Imasheva, E. S.; Yartsev, A.; Sundström, V.; Lanyi, J. K. Femtosecond Carotenoid to Retinal Energy Transfer in Xanthorhodopsin. *Biophys. J.* **2009**, *96*, 2268–2277.

(S3) Billsten, H. H.; Bhosale, P.; Yemelyanov, A.; Bernstein, P. S.; Polívka, T. Photophysical Properties of Xanthophylls in Carotenoproteins from Human Retina. *Photochem. Photobiol.* **2007**, *78*, 138–145.

(S4) Josue, J. S.; Frank, H. A. Direct Determination of the S_1 Excited-State Energies of Xanthophylls by Low-Temperature Fluorescence Spectroscopy. *J. Phys. Chem. A.* **2002**, *106*, 4815–4824.

(S5) Khokhlov, D.; Belov, A. Low-lying excited states of natural carotenoids viewed by ab initio methods. *J. Phys. Chem. A.* **2022**, *126*, 4376–4391.
